# Synthesis and Properties of the Ba_2_PrWO_6_ Double Perovskite

**DOI:** 10.1021/acs.inorgchem.4c00567

**Published:** 2024-05-20

**Authors:** Damian Wlodarczyk, Mikolaj Amilusik, Katarzyna M. Kosyl, Maciej Chrunik, Krystyna Lawniczak-Jablonska, Hanka Przybylinska, Paulina Kosmela, Michal Strankowski, Lev-Ivan Bulyk, Volodymyr Tsiumra, Rajibul Islam, Carmine Autieri, Fei Xue, Marcin Zajac, Anastasiia Lysak, Roman Minikayev, Michal Bockowski, Andrzej Suchocki

**Affiliations:** † Institute of Physics, Polish Academy of Sciences, Aleja Lotnikow 32/46, PL-02668 Warsaw, Poland; ‡ Institute of High Pressure, Polish Academy of Sciences, Sokołowska 29/37, PL-01142 Warsaw, Poland; § Military University of Technology, Gen. Sylwestra Kaliskiego 2, PL-00908 Warsaw, Poland; ∥ Gdansk University of Technology, G. Narutowicza 11/12, PL-80233 Gdansk, Poland; ⊥ Department of Physics, University of Alabama at Birmingham, Second Avenue 1720, South Birmingham, 35294 Alabama, United States; # Consiglio Nazionale delle Ricerche CNR-SPIN, UOS Salerno, C. S. V. Ferreri 12, Fisciano, IT-84084 Salerno, Italy; ¶ Solaris Synchrotron NSRC, Jagiellonian University, Czerwone Maki 98, PL-30392 Cracow, Poland

## Abstract

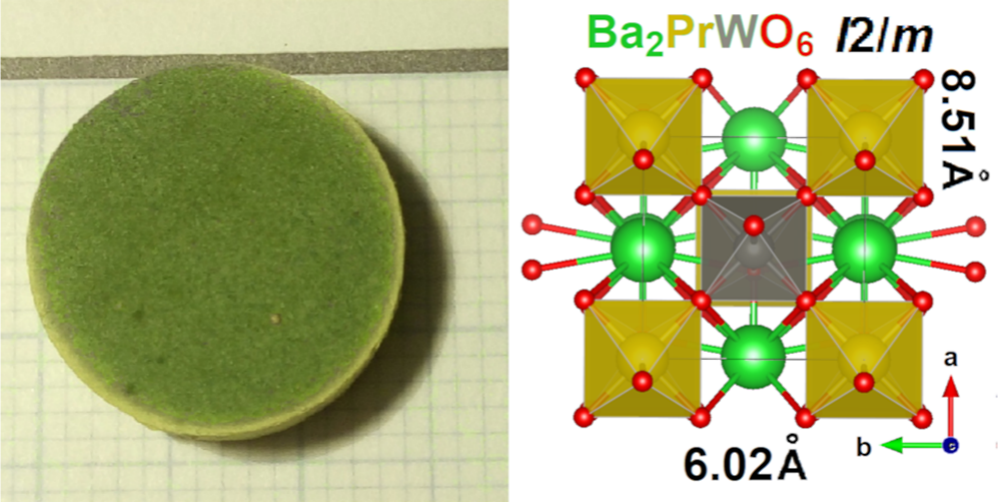

We report details on the synthesis and properties of barium praseodymium tungstate, Ba_2_PrWO_6_, a double perovskite that has not been synthesized before. Room-temperature (RT) powder X-ray diffraction identified the most probable space group (SG) as monoclinic *I*2/*m*, but it was only slightly distorted from the cubic structure. X-ray photoelectron spectroscopy confirmed that the initial (postsynthesis) material contained praseodymium in both 3+ and 4+ charge states. The former (Pr^3+^) disappeared after exposure to UV light at RT. Photoluminescence studies of Pr^3+^ revealed that Ba_2_PrWO_6_ is an insulator with a band gap exceeding 4.93 eV. Pressure-dependent Raman spectroscopy excluded the possibility of a phase transition up to 20 GPa; however, measurements between 8 and 873 K signified that there might be a change toward the lower symmetry SG below 200 K. Electron paramagnetic resonance spectra revealed the presence of interstitial oxygen which acts as a deep electron trap.

## Abstract 2

Newly synthesized Ba_2_PrWO_6_ double perovskite: an image of the pellet and unit cell visualization extracted from the refined CIF data file.

## 1 Introduction

Double perovskites (DPs) are one of the most abundant and most intensely investigated compounds.^1−4^ They have the typical ABX_3_ perovskite structure but with lattice constants doubled along all the three dimensions, creating a compound of the general A_2_BB′X_6_ formula. In this structure, the larger A cation, typically an alkali or alkaline earth metal, is 12-fold coordinated with X anions and occupies the void between corner-sharing BX_6_ and B′X_6_ octahedra. The alternating B and B′ occupancy in the adjacent octahedra is frequently referred to as a rock-salt-type arrangement. The smaller B, B′ cations are mostly different transition metal (TM) ions or combinations of rare-earth and TM ions, respectively. The X anions are usually halogens or chalcogens, but the most numerous are oxides. Depending on the kind of incorporated ions, DPs can exhibit diverse electrical or magnetic properties. Most of them are insulators; however, many are conducting, and some even show spin-dependent transport^5,6^ or superconductivity.^7−9^ Those containing paramagnetic ions can exhibit long-range magnetic order:^10,11^ ranging from antiferromagnetic,^12,13^ through ferrimagnetic,^3,14^ to ferromagnetic.^3^ Spin glass behavior is also frequently encountered.^15,16^

Perovskites gained great scientific and technological interest as microwave dielectrics,^2,17,18^ piezoelectric sensors,^2,3,19^ electrodes for fuel cells,^20−22^ magnetic memory components,^23^ catalysts,^24−26^ or solar cell materials.^27−29^

Recently, we have added a new DP, Ba_2_CeWO_6_ (BCW),^30^ to the about thousand DP oxides listed in ref (1), which were synthesized up to 2015. Now, we add another new DP to the list—Ba_2_PrWO_6_ (BPW). The replacement of Ce by Pr should improve the luminescent properties of the material and make it more suitable for prospective applications as a UV to visible light downconverter. Although the theoretically predicted compound’s structure has been reported in the Materials Project database (entry mp-1519191) up to date, there are no published experimental data, neither on the crystal structure nor on other properties, implying that the material has not been synthesized before.

This work focuses on the structural and luminescent properties of Ba_2_PrWO_6_, especially the charge-transfer processes occurring under UV illumination. Various X-ray techniques were applied, such as powder X-ray diffraction (XRD), X-ray photoelectron spectroscopy (XPS), and X-ray absorption spectroscopy (XAS), to establish the space group (SG) and charge states of the constituting ions, as well as eventual impurity phases. The results of complementary Raman and Fourier transform infrared (FTIR) studies and electron paramagnetic resonance (EPR) spectra are also reported. Since perovskites may degrade under extreme conditions,^31,32^ we also checked the stability of BPW at high pressures and high temperatures, which is discussed in more detail in the Supporting Information.

## 2 Experimental Section

### 2.1 Characterization

Scanning electron microscopy (SEM) was performed on a Hitachi SU-70 SFE device. The distribution of crystallite sizes was analyzed with ImageJ software. Powder XRD (X’pert MPD, Panalytical) was performed in Bragg–Brentano geometry with 0.05° resolution using the Cu K_α_ line. FullProf Suite was used for Rietveld refinement. XAS data were collected on pellets at the BL04 beamline of the SOLARIS NSRC synchrotron facility in Cracow, Poland. The detector was set to the total electron yield mode. XPS measurements were performed on powders placed in a PREVAC setup equipped with a Scienta R4000 200 eV hemispherical analyzer and a monochromatic Al K_α_ (∼1486.7 eV) X-ray tube. The spectra were analyzed using CASA XPS Software package version 2.3.17. Shirley background correction was used, and peaks were fitted with mixed Gaussian–Lorentzian functions. Atomic content was determined with accuracy not lower than 0.5% for W and 1.1% for Pr. EPR spectra were acquired using an X-band Bruker EMX spectrometer. Powders were placed in quartz tubes. Raman spectra were registered on a S&I Gmbh MonovistaCRS+ spectrometer equipped with a 0.75 m Acton-Princeton monochromator, a nitrogen-cooled CCD camera with a back-thinned PyLoN system, an Olympus XYZ IX71 inverted stage Moticam camera, a long working distance of 50× objective (NA 0.9), and a diode-pumped Cobolt Samba 04-01 laser emitting at 532 nm. The spectral resolution was approximately 0.88 cm^–1^. The inert N_2_ atmosphere was controlled by using the LINKAM FTIR600 stage. Samples were usually placed on (pellets) or sandwiched between (powders) thin quartz plates. FTIR spectra were registered using a Nicolet 8700 spectrometer from Thermo Electron Corp. operating in the attenuated total reflectance mode. Room-temperature (RT) photoluminescence (PL) and photoluminescence excitation (PLE) spectra were collected with a Horiba/Jobin-Yvon Fluorolog-3 spectrofluorometer using a xenon lamp as an excitation source. The excitation light was directed through a single-grating monochromator, and the emission was collected through a double-grating monochromator and recorded with a Hamamatsu R928P PMT detector. Low-temperature (LT) PL was excited with a 212 nm line of an EKSPLA NT342/3 optical parametric oscillator (OPO), dispersed through an Acton SpectraPro SP-2500 triple-grating spectrometer (Princeton Instruments), and detected with a Hamamatsu S10141 CCD area image sensor. A 435 nm filter was used to cut off the second harmonics of the laser. High hydrostatic pressure (HP) measurements were performed up to 20 GPa in an Almax easyLab diamond anvil cell. The pressure transmitting medium was argon, and ruby was used as a pressure gauge. Gaskets with 0.15 mm holes were made from Inconel ×750 alloy and filled up to 50% with powders. All LT experiments, except EPR, were performed using a continuous, helium-flow Oxford Instruments cryostat model CF1204. The achieved vacuum did not exceed 2 × 10^–5^ to 8.6 × 10^–6^ mbar. In EPR, an Oxford Instruments 910 He flow cryostat was used.

### 2.2 Synthesis

The feasibility of Ba_2_PrWO_6_ (BPW) formation was checked by calculating the classic Goldschmidt (*t*)^33^ and recently modified (τ)^34^ tolerance factors, shown in Table 1. Although the parameters for both Pr^4+^/W^4+^ and Pr^3+^/W^5+^ lie in the tolerance range between 0.825 and 1.059, the one for the former pair is closer to the ideal value of *t* = 1. The obvious choice would be to take as Pr and W sources PrO_2_ and WO_2_ oxides, but the former is stable only under high oxygen pressure. Therefore, we decided to use the naturally occurring mixed valence Pr_6_O_11_ oxide instead of Pr_2_O_3_ to improve the double-perovskite formation. The advantage is that we can retain some of the luminescent Pr^3+^ ions in the perovskite. To compensate for the extra charge, W_18_O_49_ was included by controlled preheating of WO_2_.

**Table 1:** Classic Goldschmidt (*t*) and Modified (τ) Tolerance Factors Predicting the Existence of Ba_2_PrWO_6_ Depending on the Pr and W Charge States^a^

ionic radii [Å]^35^				*t*	τ
^XII^A-site	^VI^B-site	^VI^B′-site	^II^X-site		
Ba^2+^ 1.61	Pr^4+^ 0.99	W^4+^ 0.66	O^2–^ 1.35	0.962	3.474
	Pr^3+^ 1.13	W^5+^ 0.62		0.941	3.578

a *t* = ; τ = ]; *r*_*I*_—ionic radii; *r*_*BB*′_ = 1/2(*r*_*B*_ + *r*_*B*′_).

The initial substrates used for solid-state reaction synthesis were BaCO_3_ (99.9%, Strem Chemicals) preheated to remove water, WO_2_ (99.9%, Merck), and Pr_6_O_11_ (99.99%, Strem Chemicals) taken in a weight ratio of 1.135:0.621:0.49 g (per 2 g of the final product) to ensure Ba, Pr, and W cations stoichiometry. However, XRD measurements revealed that the nominally 99.99% pure Pr_6_O_11_ phase contained 6% of Pr_3_O_5_. Prior to synthesis, WO_2_ was heated in Ar for 5 h at 650 °C. XRD performed immediately afterward identified that in the intermediate product, only 24% of tungsten was in the W_18_O_49_ phase (ICDS 05-0393 card). All substrates were then mixed in an agate mortar and compressed to a pellet with a hydraulic press at about 10 MPa.

Ba_2_PrWO_6_ was synthesized by a solid-state reaction in a quasi-inert Ar/H_2_ (999–995:1–5 mL) gas mixture. This atmosphere was essential because any surplus of oxygen redirected the reaction toward other tungstate phases. The synthesis occurred in three stages. In the first stage, the pellet was heated at 1000 °C for 10 h in a constant-flow, tube furnace under a gas pressure of 0.7 atm in order to release CO_2_. In the next stage, the temperature was increased to 1100 °C, while the duration time and gas pressure remained the same. In the last stage, the material was heated at 1100 °C for 12 h in a chamber pressurized up to ∼200 atm in order to improve the ionic migration and fusion rate.^36^ Between these stages, all pellets were ground into a fine powder and compressed back into pellets. To avoid carbon contamination, corundum crucibles were used.

## 3 Results and Discussion

### 3.1 Macro- and SEM Microphotographs

**Figure 1:**
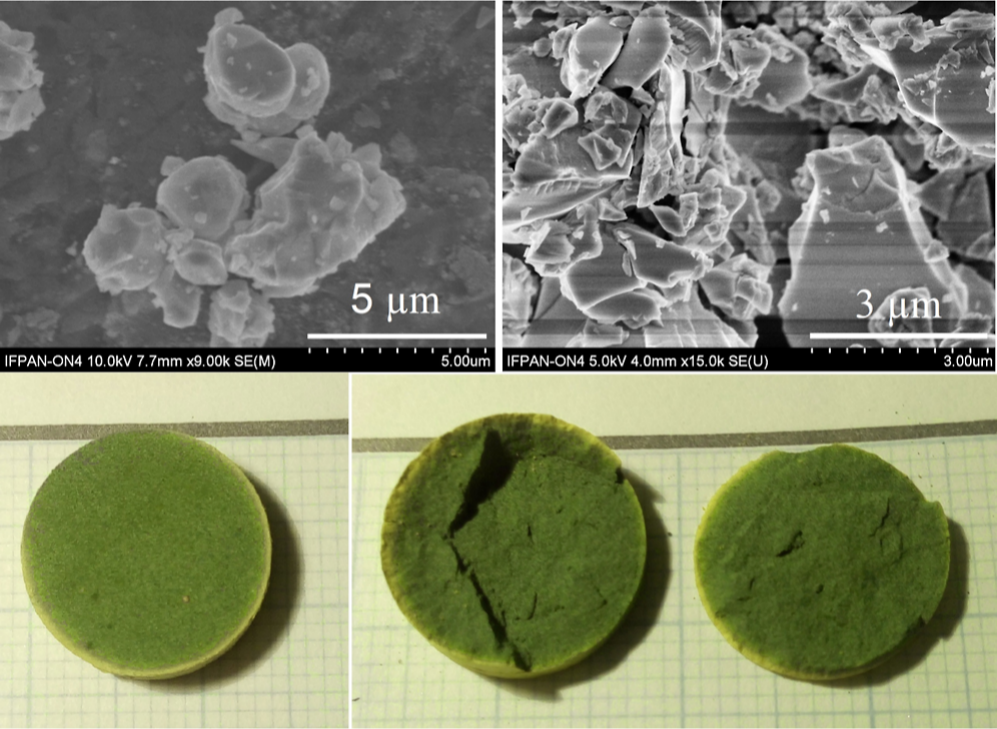
Upper panel: SEM micrographs of BPW were obtained for two different magnifications. The scale is given in the graphs. Lower panel: photographs of the obtained pellets: whole (left) and cut through (right).

SEM micrographs and photographs of the obtained material are shown in Figure 1. The lighter-colored outer rim of the pine-green BPW pellet was found to contain significantly more impurity phases than the interior; therefore, it was scrapped off and discarded prior to further investigations. The material consists of ca. micron-sized, polygon-shaped crystallite grains embedded in finer powder. The average size of 20 randomly chosen grains was about 855 ± 91 nm (with a spread from 515 to 992 nm). The full width at half-maximum (fwhm) of XRD peaks can give more information on the crystallite size, according to the Debye–Scherrer equation.^37^ Although for grains larger than 200 nm the method is considered to be less accurate, it is more adequate than estimates based on particular, very small areas visualized in SEM. Based on shape constants (*K* ∼ 0.88–0.93) for the three dominant peaks at 2Θ = 110.3, 85.4, and 69.8 and their fwhm’s, the determined average size is 914 ± 7 nm.

When heated in the air, BPW slowly decomposes to an orange powder starting from the surface (Figure S1). The mechanism of oxidation can vary but mainly involves the formation of BaWO_4_, as discussed in the Supporting Information.

### 3.2 Powder XRD

**Figure 2:**
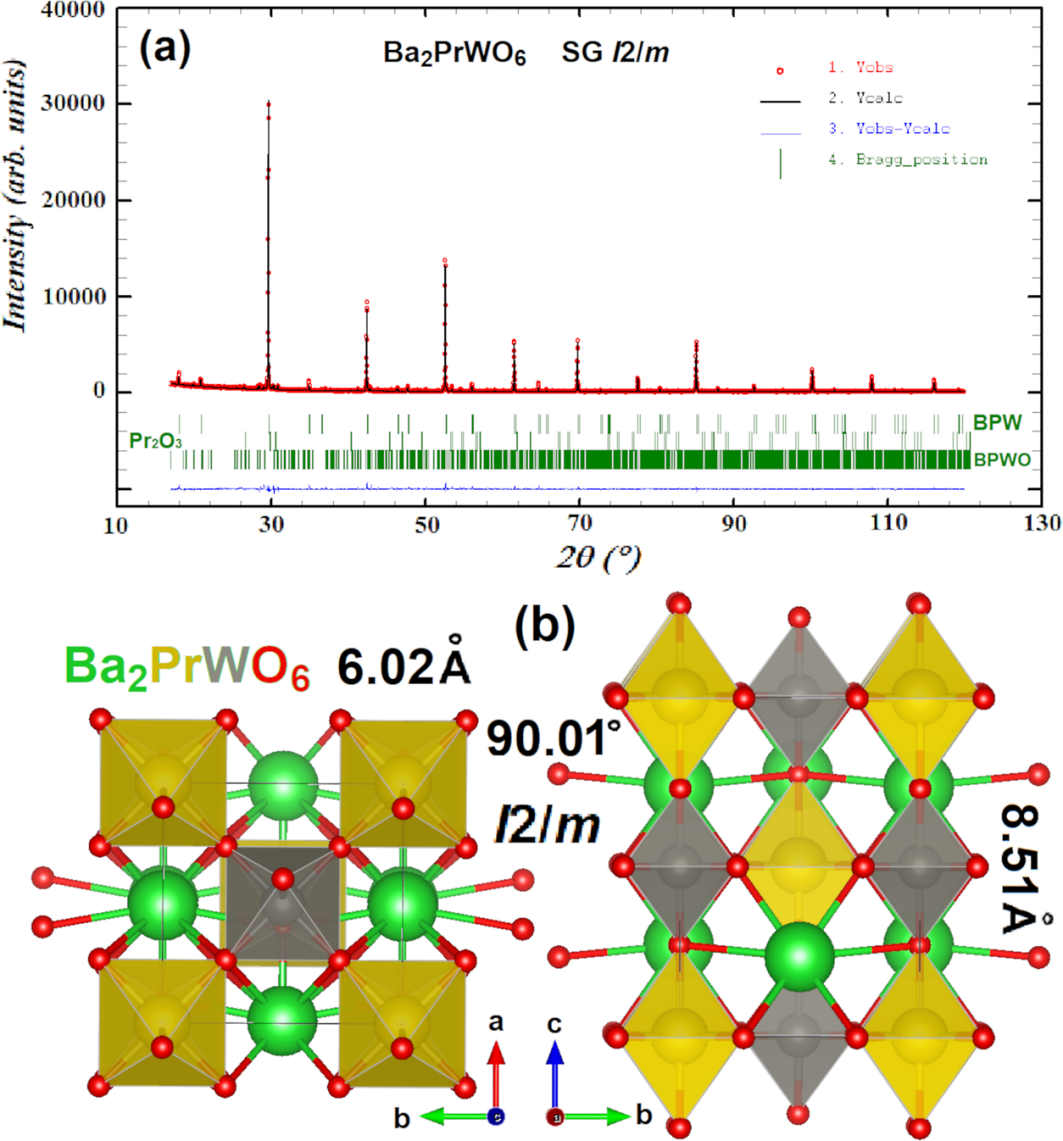
(a) XRD pattern of the synthesis product: dots—experimental data and black line—Rietveld refined fit assuming ∼96% of the Ba_2_PrWO_6_*I*2/*m* phase, ∼4% of BaPr_2_WO_7_ (BPWO), and ∼0.3% of Pr_2_O_3_. Blue line: difference between experimental and calculated data. Green bars denote the Bragg positions for the three phases. (b) Projection of the unit cell on the ab plane (left) and the bc plane (right). Green, yellow, gray, and red spheres denote Ba, Pr, W, and O ions, respectively.

The powder XRD pattern of the synthesized product is shown in Figure 2a. The best Rietveld refined fit to the experimental pattern was obtained for 95.71% Ba_2_PrWO_6_ with the monoclinic *I*2/*m* SG, similar to Ba_2_BiYO_6_ (ICSD 65555).^38^ The structure is shown in Figure 2b. The unit cell parameters are *a* = 6.0219(3) Å, *b* = 6.0218(3) Å, *c* = 8.5167(3), β = 90.01(3)°, and *V* = 308.84(3) Å^3^. The detected impurity phases are BaPr_2_WO_7_ ceramics (3.97%) with a structure similar to that of SrLa_2_WO_7_ (JCPDS 049-0353) and Pr_2_O_3_ (0.32%).^39^

A possible alternative to the *I*2/*m* SG is *R*-3, with the following unit cell parameters: *a* = *b* = 6.0219(4) Å, *c* = 14.7509(8) Å, and *V* = 463.26(1) Å^3^, for which the error factor is only slightly greater (χ^2^ = 42.2 for *I*2/*m* and 43.1 for *R*-3). The refined fit to the same XRD experimental data and the derived structure are presented in Figure S2. For the sake of further discussion, the *I*2/*m* SG is chosen; however, to confirm this assignment unambiguously, neutron powder diffraction would be necessary.

The background-corrected Rietveld refinement parameters for the alternative *R*-3 SG are given in Table S1 while those for *I*2/*m* are presented in Table 2. The atomic coordinates and occupancy factors are collected in Table 3 (and Table S2 for *R*-3).

**Table 2:** Rietveld Reliability Factors for the Diffractogram Are Given in Figure 2a

formula	SG	*Z*	*V* [Å^3^]	*d*_cal_ [g/cm^3^]	*R*_B_	*R*_p_	*R*_wp_	*R*_exp_	*N*_σ_ GoF	χ^2^	fract. [%]
Ba_2_PrWO_6_	*I*2/*m*	2	308.843	7.479	5.75	18.3	17.3	2.66	2674.274	42.2	95.71
BaPr_2_WO_7_	*P*112_1_/*b*	4	623.818	7.613	28.5						3.97
Pr_2_O_3_	*P*-3*m*	1	77.676	7.051	57.9						0.32

**Table 3:** Atomic Coordinates and Occupancy Factors for the *I*2/*m* SG

site label	*x*/*a*	*y*/*b*	*z*/*c*	occupancy
Ba1	0.514(2)	0.00000	0.2638(11)	1.00000
Pr1	0.00000	0.00000	0.00000	1.00000
W1	0.00000	0.00000	0.50000	1.00000
O1	–0.092(4)	0.00000	0.295(4)	1.00000
O2	0.282(3)	0.26300	–0.006(4)	1.00000

### 3.3RT XPS Data

XPS is a standard technique to determine the atomic content of solids. In contrast to XRD, which detects only crystalline phases, in XPS, all ions are detected, including the ones at interstitial sites or residing in amorphous phases. Another advantage of XPS is the distinction of valence states of the constituting ions. The latter can be, however, treated only as an indication since the measurement is performed under ionizing radiation, which significantly changes the charge-state occupancies. We employed this technique to determine the total atomic content in the synthesis product.

**Figure 3:**
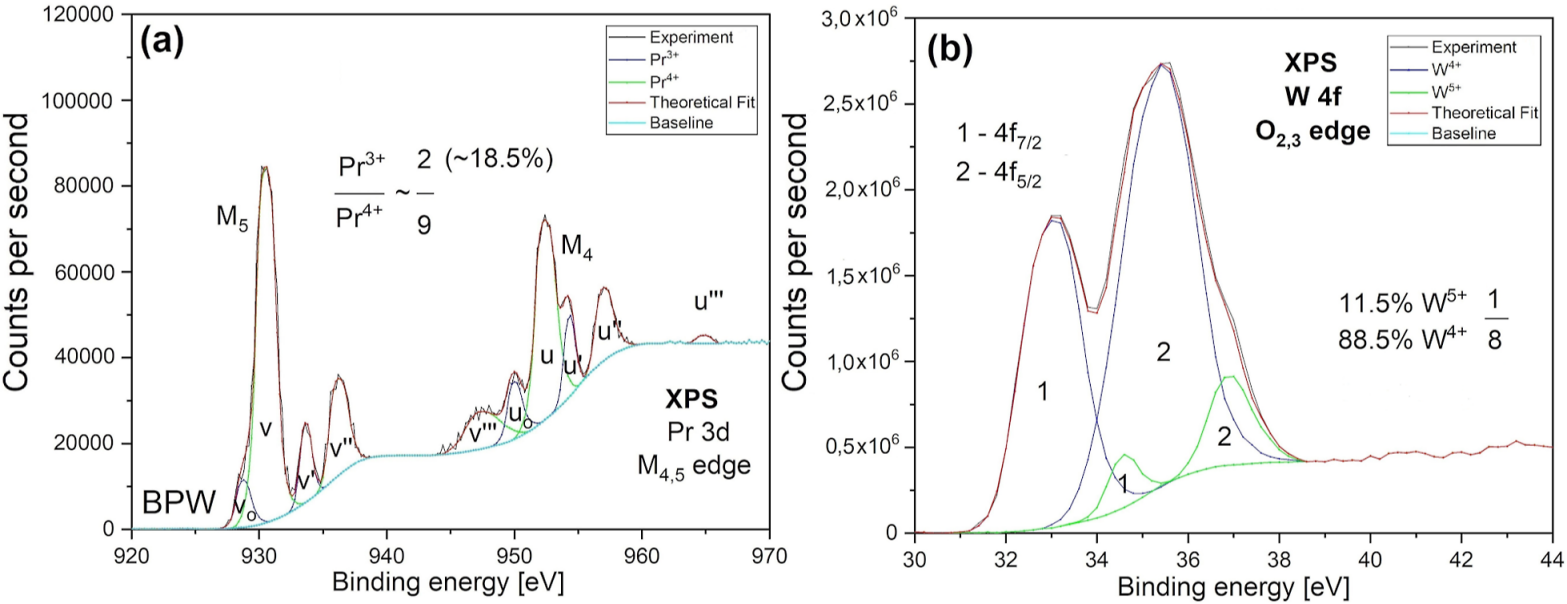
X-ray photoelectron spectra of the Pr 3d (a) and W 4f (b) core states. Red lines are fits to experimental data (black lines) taking into account two charge states for each ion (blue and green lines) and the background (cyan line).

The X-ray photoelectron spectrum of the Pr 3d_5/2_ and 3d_3/2_ spin–orbit split core levels (M_5_ and M_4_ edges, respectively) is shown in Figure 3a. The rich structure can be deconvoluted into three sets of doublets related to Pr^4+^, denoted as *v*, *v*″, and *v*‴ in the d_5/2_ component and *u*, *u*″, and *u*‴ in the d_3/2_ component—green lines in Figure 3a. The peaks denoted as *v*_o_, *v*′ (d_5/2_) and *u*_o_, *u*′ (d_3/2_), depicted by blue lines, are ascribed to Pr^3+^.^40−42^ We follow here the peak notation and assignment of Konysheva and Kuznetsov.^42^ The same peaks are detected in the X-ray absorption spectrum of BPW (Figure S3).

In contrast to praseodymium, the XPS spectrum of tungsten 4f O_2,3_ edges shown in Figure 3b contains only two spin–orbit split doublets, denoted by “1” and “2” for the 4f_7/2_ and 4f_5/2_ core states, respectively. The intense peaks at binding energies (BE) of 33.1 eV (4f_7/2_) and 35.4 eV (4f_5/2_), depicted by the blue line, are assigned to W^4+^, while those at 34.6 and 36.9 eV (green line) to W^5+^.^43,44^ No signal related to W^6+^ was detected (expected at BE = 35.6–35.7 eV),^43,44^ which indicates that the charge state occurring in the initial substrate was reduced during synthesis, most probably due to electron transfer from some of the Pr^3+^ ions.

**Figure 4:**
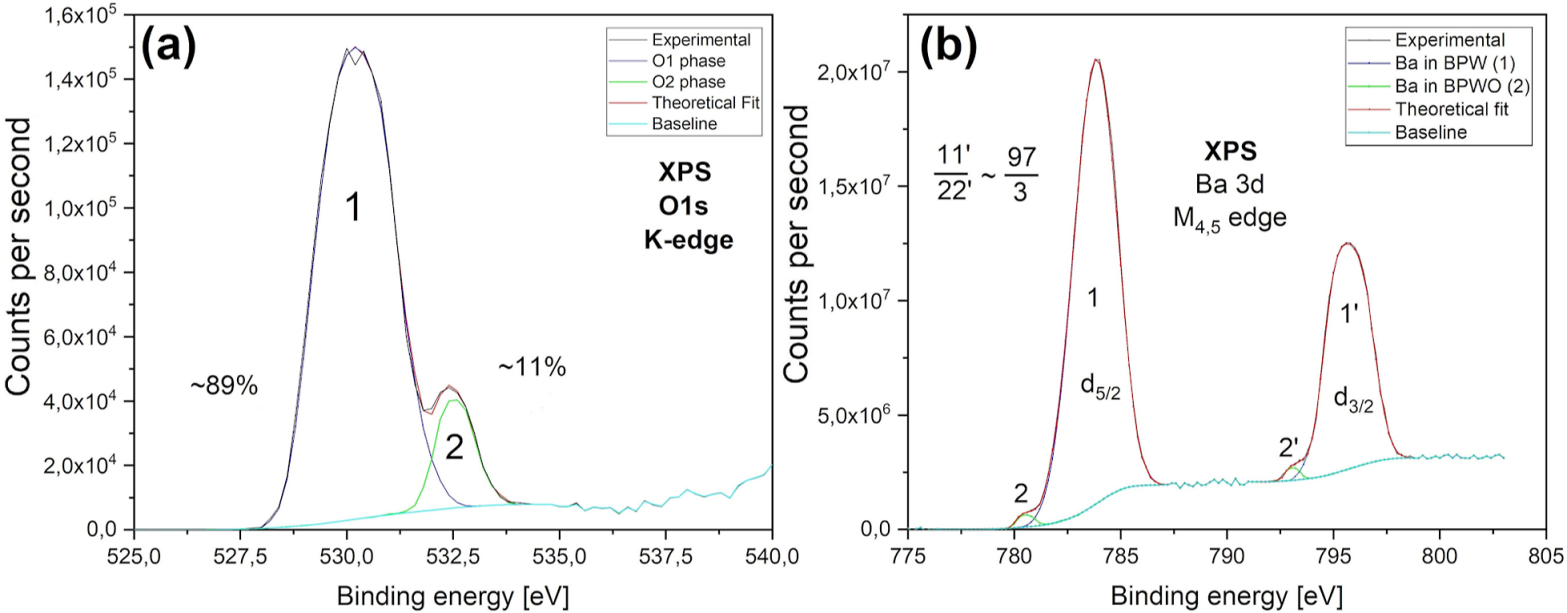
XPS spectra of the O 1s K-edge (a) and Ba 3d M_4,5_ edges (b).

The oxygen K-edge photoelectron spectrum is shown in Figure 4a. It consists of a very broad, intense peak at BE = 530.2 eV and a more than twice narrower, lower intensity peak at 532.6 eV. Since there are different surroundings of oxygen in the BPW lattice depending on the charge states of praseodymium and tungsten ions, we assign the broad peak to the substitutional oxygen and the weaker one to interstitial oxygen (O_i_). The presence of O_i_^–^ was confirmed by EPR experiments (Section 3.4). The barium M_4,5_-edge XPS spectrum presented in Figure 4b shows a strong spin–orbit split 3d_5/2_ and 3d_3/2_ doublet, located at 783.8 and 795.7 eV, respectively, which originates from the main BPW phase. The weak shoulders located at 780.5 and 793.4 eV most likely stem from the BPWO phase. The peak positions do not differ much from typical values.^45,46^

The peak positions, fwhm, and determined percentage atomic contents for all XPS-analyzed species are collected in Table 4, together with the atomic fractions normalized to tungsten content. For comparison, the atomic fractions summed for all crystalline phases detected by XRD are also given. The latter data are not much different from those obtained by XPS (also indicating a low contribution of amorphous phases), except that significantly more oxygen is detected by XPS. This supports our assignment of the 532.6 eV O 1s XPS peak to interstitial oxygen. The numbers given in Figures 3 and 4 pertain only to the initial, relative charge distribution under ionizing radiation and cannot accurately determine the true number of constituting ion states, especially considering the influence of the other phenomena, like charge transfer, described in the next section.

**Table 4:** Summary of XPS Investigations of Ionic Core Levels of BPW: Peak Positions, FWHM, Percentage Atomic Contents, and Ratios (Normalized to W) Compared to Those Determined from XRD for Detected Crystalline Phases

core level	s–o split	position [eV]	fwhm [eV]	total content [%]	ion fraction	
					XPS	XRD
W 4f	7/2	33.1; 34.6	1.76; 0.62	8.69	1	1
	5/2	35.4; 36.9	1.89; 1.13			
O 1s	–	530.2; 532.6	2.47; 1.05	62.95	7.24	6.05
Ba 3d	5/2	780.5; 783.9	0.89; 2.41	18.15	2.09	1.96
	3/2	793.1; 795.7	0.79; 3.04			
Pr 3d	5/2	928.8; 930.5; 933.6	1.32; 1.73; 1.04	10.21	1.17	1.05
		936.2; 947.5	1.61; 2.91			
	3/2	949.9; 952.4; 954.4	1.26; 1.78; 0.98			
		957.0; 964.8	1.52; 1.42			

### 3.4 Charge-Transfer Processes

PL and EPR are the easiest experimental techniques to monitor changes in the charge states of constituting ions that occur after near-UV illumination. Especially, the latter technique can provide information on the presence of additional paramagnetic defects, which can take part in charge-transfer processes. In this subsection, we seek answers to the following questions: (i) what is the mechanism of electron release from Pr^3+^? and (ii) on which ions, apart from W^5+^, are the released electrons captured?

**Figure 5:**
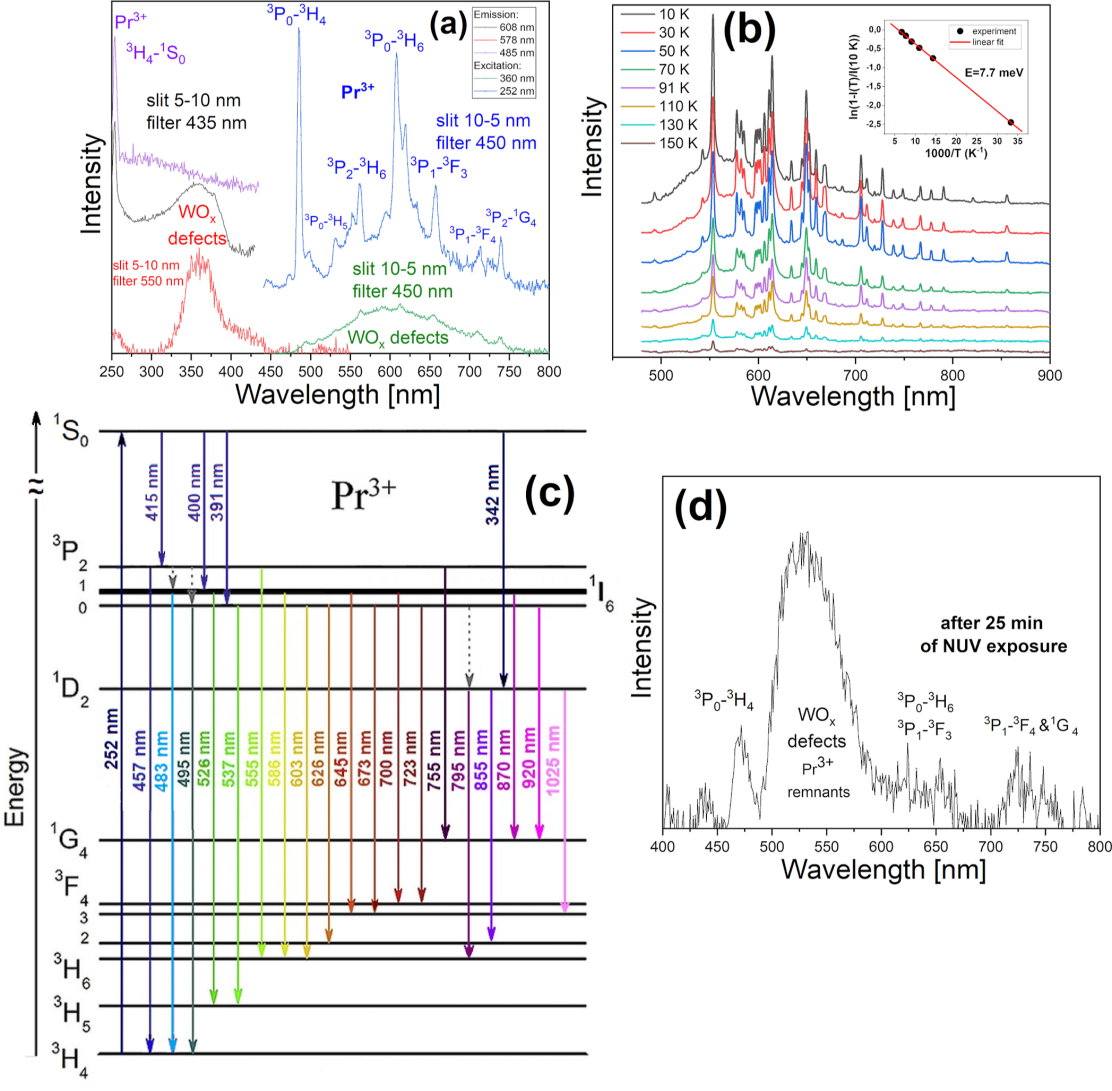
(a) PL and PLE spectra of BPW at RT. The excitation (for PL) and detection (for PLE) wavelengths are given in the legend. The spectra are shifted vertically for clarity. (b) Selected temperature-dependent spectra under 232 nm excitation of the OPO laser. The inset shows the Arrhenius plot of the integrated PL intensity loss, *I*(10 K) – *I*(*T*), normalized to the initial value at 10 K. (c) Energy level diagram of Pr^3+^. (d) RT PL after 25 min exposure to UV illumination (λ ≤ 252 nm).

The PL and PLE spectra collected at RT are shown in Figure 5a. The excitation (for PL) and detection (for PLE) wavelengths are given in the legend. As an excitation source, a xenon lamp was used. Under 252 nm excitation, the luminescence is dominated by sharp lines stemming from the characteristic intra 4f-shell transitions of Pr^3+^,^47,48^ superimposed on a weak, broad band attributed tentatively to WO_*x*_ charge-transfer transitions.^49^ The energy level diagram of Pr^3+^, together with the assignment of the observed transitions, is presented in Figure 5c. The PL excitation spectrum monitored for the strongest Pr^3+^ emission line at 485 nm shows a sharp line at 252 nm, related to intrashell ^3^H_4_ → ^1^S_0_ transition,^50^ while the spectrum detected for wavelengths between the sharp Pr^3+^ lines, e.g., 578 nm, shows a broad absorption band at about 360 nm. Excitation within this range does not lead to Pr^3+^ emission.

At RT, the Pr^3+^ PL intensity decreases strongly with the irradiation time. Already after 25 min of exposure to UV light, the luminescence is almost completely bleached, as shown in Figure 5d. In contrast, at 10 K, it remains practically stable. The results of LT PL measurements presented in Figure 5b indicate that the bleaching efficiency depends exponentially on the temperature. The deactivation energy determined from the loss of integrated intensity vs inverse temperature is equal to 7.7 meV (see the inset in Figure 5b) and corresponds to the thermal ionization energy of an electron from the ^1^S_0_ excited state. The LT PL spectrum recorded without a 435 nm cutoff filter is shown in Figure S4.

**Figure 6:**
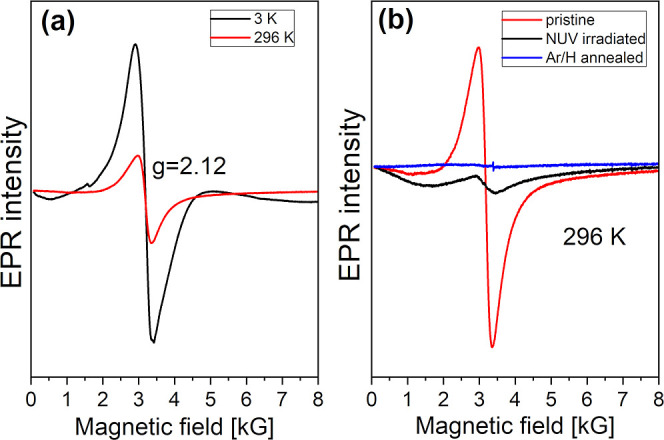
(a) EPR spectra of BPW at 3 and 296 K (black and red lines, respectively). (b) RT EPR spectra before UV irradiation (red line), after 25 min of UV irradiation (black line), and after subsequent annealing in reducing Ar/H_2_ atmosphere (blue line).

The EPR spectrum of BPW recorded at 3 K is presented in Figure 6a. Since Pr^3+^ ions with 4f^2^ electron configuration cannot be detected in our conventional EPR setup,^51^ we expected to detect mainly an anisotropic (C_2h_ local symmetry) powder spectrum of Pr^4+^ (4f^1^)^52,53^ ions, similar to that of the isoelectronic Ce^3+^ observed previously in BCW,^30^ which would be visible only at cryogenic temperatures. Instead, the spectrum is dominated by an isotropic paramagnetic signal that persists up to RT (red line in Figure 6a). The lack of fine structure and the *g*-factor of 2.12 indicate an acceptor-type defect with spin *S* = 1/2. The obvious candidate is interstitial oxygen in the −1 charge state (O_i_^–^). Substitutional O_s_^–^ can be excluded due to lower (C_s_ or C_1_) site symmetry. After UV irradiation at RT, the signal intensity is drastically reduced (compare red and black lines in Figure 6b), which indicates that a part of the electrons ionized from Pr^3+^ is captured by O_i_^–^ forming diamagnetic O_i_^2–^. The assignment of the defect to interstitial oxygen is further corroborated by the fact that annealing in a reducing atmosphere containing H_2_ completely removes the EPR signal, as shown by the blue line in Figure 6b.

The lack of an EPR signal from Pr^4+^ ions, despite their high concentration, is probably an effect of antiferromagnetic ordering at LTs since most of the A_2_BB′O_6_ perovskites containing a single paramagnetic B site cation are reported to be antiferromagnetic (Vasala and Karppinen^1^ and references therein). In BCW, most of the ceria were in the diamagnetic 4+ charge state (4f^0^);^30^ thus, the distance between paramagnetic Ce^3+^ ions was too large for superexchange interaction to be effective and the compound remained paramagnetic even at 3 K.

**Figure 7:**
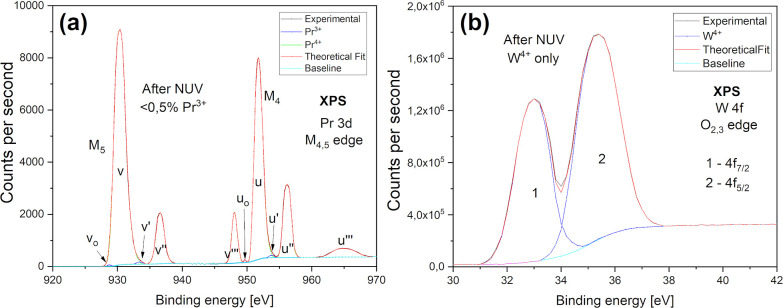
XPS spectra of Pr 3d (a) and W 4f (b) core states recorded after 25 min of exposure to UV illumination.

The photoelectron spectra of Pr 3d and W 4f core levels presented in Figure 7 show that after exposure to a broad-spectrum UV light source, all of the tungsten and practically all praseodymium ions (except a trace fraction) occur in the 4+ charge state. The fact that both W^5+^ and O_i_^–^ capture electrons thermally ionized from the ^1^S_0_ excited state of Pr^3+^ proves that the charge-transfer process occurs via the conduction band. It allows us, moreover, to place the energy level of the ^3^H_4_ ground state of Pr^3+^ about 4.93 eV below the minimum of the BPW conduction band. This value is considerably larger than the band gap obtained from density functional theory (DFT) calculations (Figure S5); however, it is well known that DFT drastically underestimates band gaps. The situation changes further only for tungsten if the sample is carelessly exposed to a more intense, laser light from UV–C spectrum without any protective atmosphere. Special care must be taken since even O_i_ is prone to react if the sample is luminously overstimulated—more about this is in Supporting Information Section 3.4 page S8.

### 3.5 Raman and FTIR Spectroscopies

**Figure 8:**
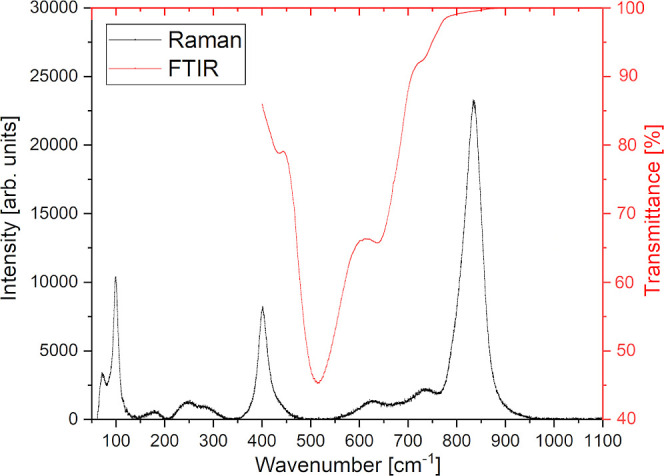
Raman (black line, left axis) and FTIR (red line, right axis) spectra of BPW recorded under ambient conditions. No signals were detected above 1100 cm^–1^ (up to 4000 cm^–1^).

Raman and FTIR experiments were performed as an attempt to clear up doubts about the proper choice of the BPW SG (*I*2/*m* or *R*-3). The spectra recorded under ambient conditions are compared in Figure 8. The former was recorded on a single-crystalline grain, hoping to minimize the peak widths. The deconvoluted peak positions, together with literature-based mode assignments,^54−58^ are summarized in Table 5. As can be seen, the number of detected Raman peaks exceeds the 12 modes predicted by group theory for perfect *I*2/*m* (7A_1g_ + 5B_g_) as well as *R*-3 (4A_g_ + 4^1^E_g_ + 4^2^E_g_) SGs (for the total number of predicted phonon modes and their irreducible representations, see Table S3). This may be due to different Pr^3+/4+^/W^4+,5+^ ion pairings and the presence of point defects. Unfortunately, not only XRD patterns but also Raman data cannot unambiguously distinguish between *I*2/*m* and *R*-3 SGs. Also, the FTIR spectrum does not give much support since the signals are quite broad (fwhm ∼ 64 cm^–1^) and cut off below 400 cm^–1^.

**Table 5:** Experimental Raman and FTIR Data Obtained at Ambient Conditions, with Literature-Based Phonon Assignments^30,54−58^^a^

Raman modes		IR modes	
ω_0_ (cm^–1^)	assignment	ω_0_ (cm^–1^)	assignment
72.2	A-site lattice translations of mixed B_g_ and ^1^E_g_/^2^E_g_ origin	435	in-plane and out-of-plane bending MO_6_ bands of B_u_/E_u_ origin down to 100 cm^–1^
99.5		514.5	ν_sym_ stretch of MO_6_ octahedra of A_u_ origin
163.1		639	ν_asym_ stretch of MO_6_ octahedra of A_u_ origin
179.8	in-plane σ_ρ_ and out-of-plane σ_τ_ bending in MO_6_ octahedra of mixed B_g_ and ^1^E_g_/^2^E_g_ origin	731.5	
*247.4		834	
280.0			
315.7			
367.1	in-plane σ_sc_ and out-of-plane σ_ω_ bending in MO_6_ of mixed B_g_ and ^1^E_g_ origin		
401.2			
434.8			
*458.2	ν_sym_ mode of PrO_2–*x*_, (F_2g_)		
*564.4	transverse MO_6_ motion of mixed A_1g_ and A_g_ character		
625.8			
678.7			
734.5	ν_asym_ and ν_sym_ oxygen stretches of A_1g_ or A_g_ symmetry for various Wyckoff sites not only partially occupied but also hosting B-site Pr/W ions with different charges		
791.3			
835.1			
851.6			

a Asterisks mark modes that could also be associated with PrO_2–*x*_ impurities according to theoretical calculations.^59^ ν_sym_—symmetric stretching; ν_asym_—asymmetric stretching; σ_sc_—scissoring deformation; σ_ω_—wagging deformation; σ_τ_—twisting deformation; σ_ρ_—rocking deformation; and M—Pr or W ions.

**Figure 9:**
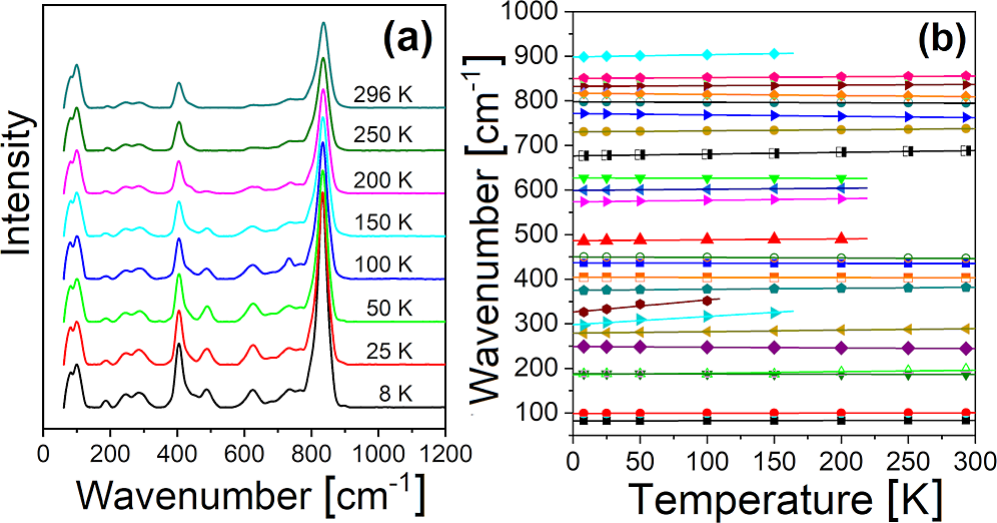
(a) Temperature-dependent Raman spectra. (b) Raman peak positions vs temperature (symbols). Linear fits are depicted with solid lines.

To shed more light on the proper SG choice, Raman studies at non-ambient conditions were performed, in order to search for possible phase transitions. These usually follow a specific SG sequence, which can indirectly point to the most probable initial SG.^1,60,61^ Raman spectra recorded at LTs (increasing from 8 K to RT) are presented in Figure 9a, and the temperature dependence of the peak positions is given in Figure 9b. It can be seen that the 8 K spectrum contains 7 peaks more than the RT one.

**Figure 10:**
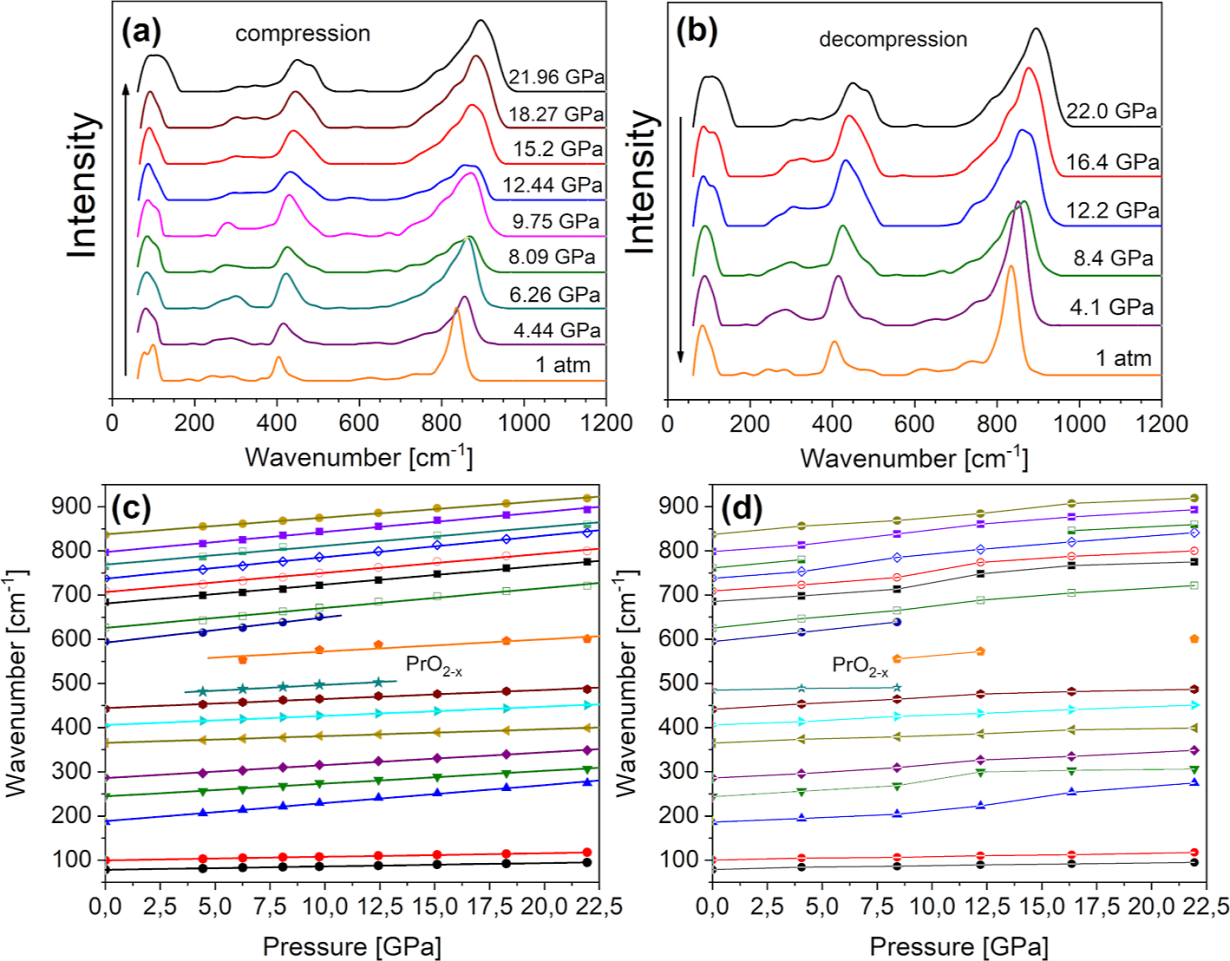
Raman spectra collected under HP by using consecutive compression (a) and decompression (b) cycles. Peak positions (symbols) vs increasing (c) and decreasing (d) pressure. Solid lines in (c) are linear fits and in (d) are guides for the eye.

Four (out of seven) of the additional peaks disappear above 150 K, and their positions change with higher temperature coefficients than those of the other three, which remain detectable until 200 K. The peak positions and temperature coefficients are collected in Table 6. The greater number of observed Raman modes may indicate a phase transition to a group with a lower symmetry (possibly monoclinic *P*2_1_/*n*). It is tempting to associate the apparent symmetry lowering either with induced magnetic ordering or with increasing octahedral tilt,^61^ however, some of the extra modes are observed under residual Ar pressure at RT, as shown in Figure 10.

**Table 6:** Raman Modes at 296 K (ω_RT_), 8 K (ω_LT_), and Temperature Coefficients (dω_LT_/d*T*)^a^

ω_RT_ (cm^–1^)	ω_LT_ (8 K) (cm^–1^)	dω_LT_/d*T* (cm^–1^/K)	ω_P_ (cm^–1^)	dω_P_/d*P* (cm^–1^/GPa)
83.7	82.4	0.0044 ± 0.0002	78.3	0.76 ± 0.01
100.3	99.1	0.0041 ± 0.0001	99.1	0.82 ± 0.02
186.5	188.2	–0.0058 ± 0.0003	186.2	4.08 ± 0.10
198.2	188.2	0.034 ± 0.008		
244.6	249.0	–0.0149 ± 0.0003	244.4	2.89 ± 0.07
288.8	278.6	0.034 ± 0.002	287	2.91 ± 0.08
	297.1	0.18 ± 0.02		
	326.2	0.27 ± 0.06		
381.7	374.7	0.023 ± 0.002	365.4	1.55 ± 0.03
403.3	404.5	–0.0041 ± 0.0002	404.5	2.07 ± 0.05
435.1	436.6	–0.0054 ± 0.0002		
446.7	450.1	–0.0118 ± 0.0004	442.2	2.06 ± 0.09
	485.9	0.020 ± 0.004	481.2 (4.44 GPa)^b^	2.6 ± 0.2
	573.5	0.035 ± 0.001	553.6 (6.26 GPa)^b^	2.8 ± 0.7
	599.4	0.024 ± 0.002	594.5	5.7 ± 0.4
	627.0	–0.0039 ± 0.0004	626.1	4.52 ± 0.13
688.0	676.6	0.040 ± 0.002	684	4.30 ± 0.10
			709.8	4.35 ± 0.13
737.5	730.6	0.0241 ± 0.0004	737.7	4.86 ± 0.10
762.8	771.6	–0.0305 ± 0.0005	764.9	4.26 ± 0.22
794.8	798.0	–0.0107 ± 0.0004	796.4	4.54 ± 0.12
809.4	817.0	–0.0107 ± 0.0004		
836.7	832.6	0.0140 ± 0.0002	836.2	2.06 ± 0.09
855.6	850.3	0.0180 ± 0.0003		
	898.4	0.0495 ± 0.0032		

a RT peak positions in Ar atm (ω_P_) and their pressure coefficients (dω_P_/d*P*) up to 20 GPa.

b Presumably PrO_2–*x*_.

RT Raman spectra under compression and decompression cycles are presented in Figure 10a,b, respectively, while the peak positions vs increasing and decreasing pressure are depicted in Figure 10c,d. Typical linear blue shifts of the Raman modes are observed with increasing pressure, but no phase transition occurs up to 20 GPa. The pressure coefficients are given in Table 6. Some peaks are so weak that their pressure dependence cannot be followed in the whole range—this concerns especially the debatable ones related to PrO_2–*x*_ defects (see Figure S6). Phase transitions at higher pressures cannot be excluded, but up to 20 GPa, the material is quite stable as hysteresis during pressure release is not higher than 2 GPa, comparable to BCW from our previous work.^30^

### 3.6 Material Stability at High Temperatures

The stability of BPW at high temperatures is important from the point of view of possible applications, e.g., as a down-converter in solar panels. Therefore, XRD and Raman studies were performed up to 600 °C in both air and inert N_2_ gas. The results of differential scanning calorimetry (DSC), thermogravimetric analysis (TGA), and heat capacity measurements performed in a similar temperature range are shown in Figure S7.

**Figure 11:**
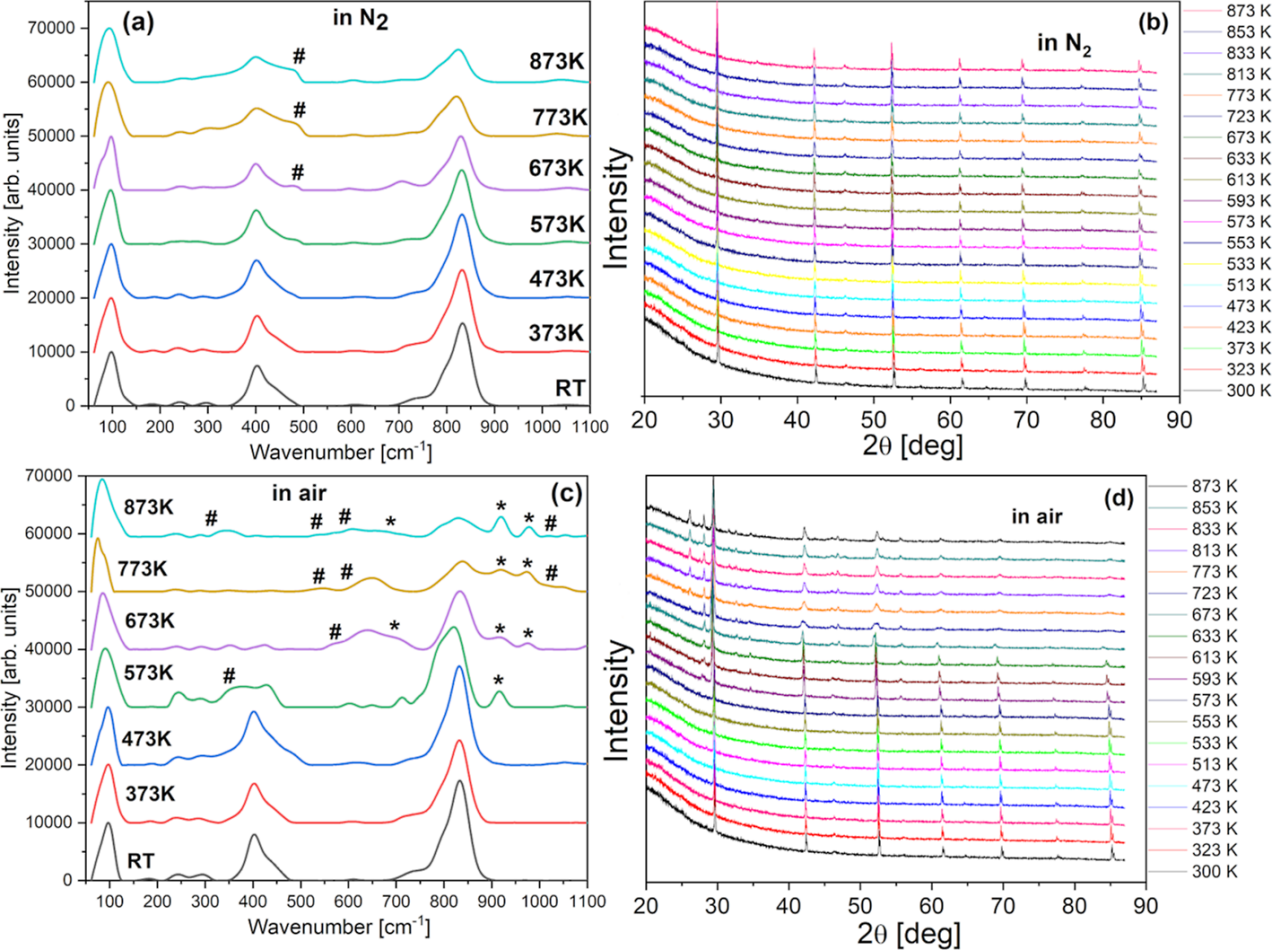
Temperature-dependent Raman spectra (a,c) and powder XRD (b,d) patterns recorded when heated in an inert N_2_ atmosphere (a,b) and in air (c,d).

Temperature-dependent Raman spectra and XRD patterns of BPW are presented in Figure 11. When heated in a protective N_2_ atmosphere (Figure 11a,b), no significant changes in the spectra were observed up to 873 K. In the Raman spectra (Figure 11a), a barely noticeable red shift can be seen with some intensity increase of PrO_2–*x*_ modes above 600 K. The XRD peaks (Figure 11b) also shift slowly with increasing temperature, indicating lattice expansion and changing tilt angle, yet no structural transition occurs. In air, the situation is totally different. Above 500 K, the Raman spectra as well as the XRD patterns visibly change (Figure 11c,d). In Raman spectra, modes related to Ba_*x*_WO_3+*x*_ and PrO_2–*x*_ species (marked by asterisks and hashtags in Figure 11c, respectively) gradually appear, indicating decomposition of BPW. The same is visible in XRD patterns. The peaks related to BPW broaden and become weaker, while new sharp peaks, related mainly to Pr_2_O_3_, BaWO_4_, and BaPr_2_WO_7_, gain intensity.

**Figure 12:**
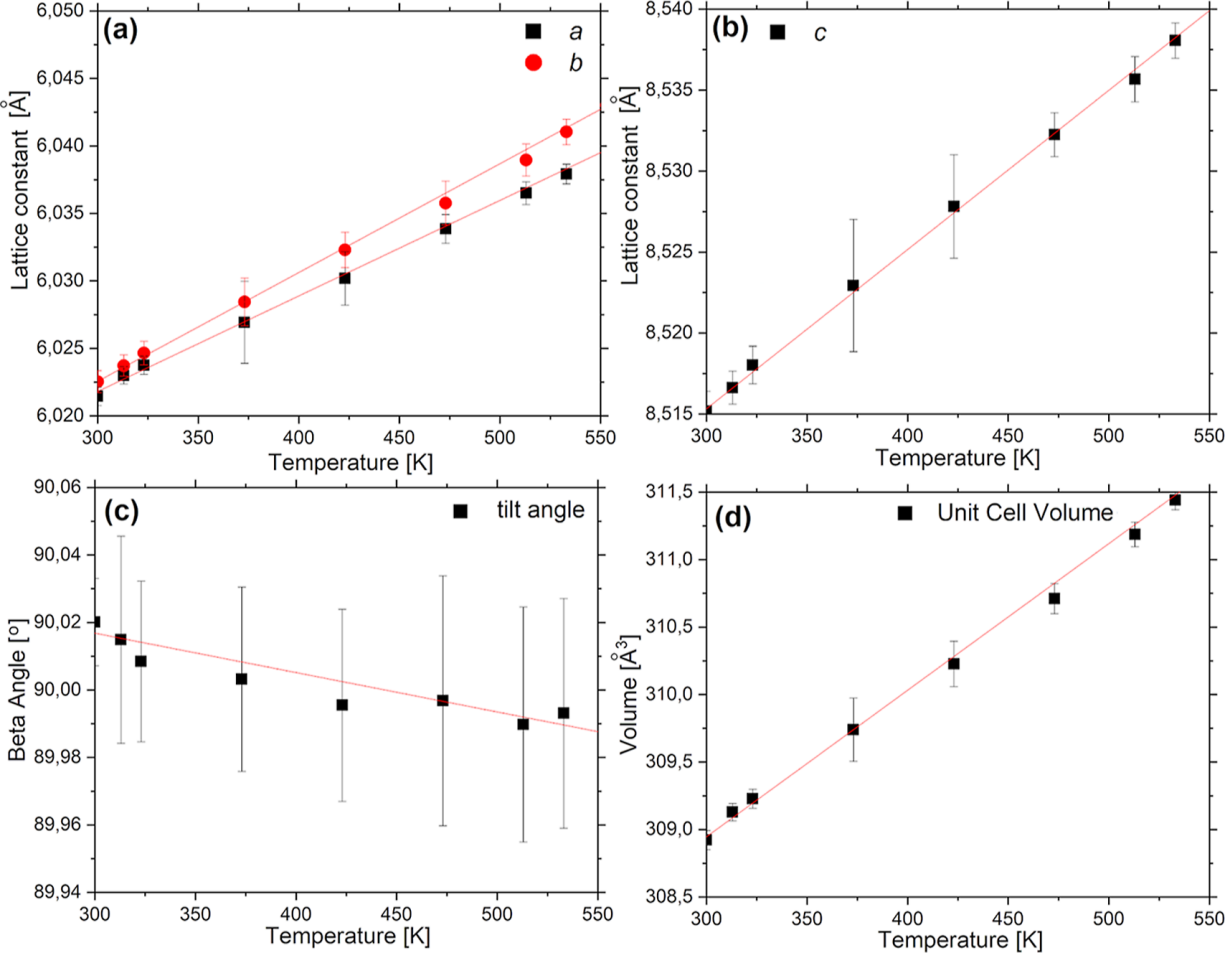
Temperature dependencies of lattice parameters: (a) *a* and *b* (black and red symbols, respectively), (b) *c*, and (c) β tilt angle. (d) Temperature dependence of the unit cell volume. Solid lines are linear fits.

The temperature dependencies of the unit cell parameters determined from XRD measurements (performed in the air) in the 300–550 K temperature range, i.e., before any signs of decomposition were noted, are shown in Figure 12. The determined temperature coefficients for the *a*, *b*, and *c* lattice constants are 7.08(0) ± 0.17(2) × 10^–5^, 8.06(5) ± 0.16(3) × 10^–5^, and 9.83(3) ± 0.23(2) × 10^–5^ Å/K, respectively. The tilt angle decreases at a rate of −1.16(7) ± 0.10(5) × 10^–4^ deg/K, and the unit volume expands at a rate of 0.0108(6) ± 0.0002(7) Å^3^/K.

## 4 Conclusions

The newly synthesized ordered barium praseodymium-tungstate double-perovskite Ba_2_PrWO_6_ (BPW) is reported. XRD studies have shown that the crystal structure of BPW (∼96% pure) is better described by the SG (*a*^0^*b*^–^*b*^–^) *I*2/*m* with *a* = 6.0219(3) Å, *b* = 6.0218(3) Å, *c* = 8.5167(3) Å, and slightly tilted β angle ∼90.01(0)° (*R*_wp_ ∼ 17.3%) than by *R*-3 with *a* = *b* = 6.0219(4) Å and *c* = 14.7509(8) Å (*R*_wp_ ∼ 17.5%). The greater number of Raman modes obtained at LTs than at RT could point to the possible onset of a transition to a lower symmetry (*P*2_1_/*n*) phase. This would favor the *I*2/*m* SG assignment, according to group theory considerations, since an *R*-3—*I*2/*m* phase transition would not increase the number of modes (Figure S8 and Table S4). Still, this assignment is not really unambiguous. With increasing temperature, the Raman lines considerably broaden, and some of them can no longer be detected. CIF structure details for both SGs are provided in Supporting Information Table S5.

XPS investigations have shown that the freshly acquired material contains Pr^3+^ and Pr^4+^ ions as well as W^5+^ and W^4+^. The estimated total atomic content makes the material stoichiometrically closer to Ba_2.09_Pr_1.17_WO_7.24_ than XRD-determined Ba_1.96_Pr_1.05_WO_6.05_ (almost ideal Ba_2_PrWO_6_). However, one has to bear in mind that in the former formula, the presence of impurity phases (∼4% BaPr_2_WO_7_ and ∼0.3% Pr_2_O_3_) as well as interstitial oxygen ions (unambiguously detected by EPR) is taken into account.

The Pr^3+^ content was shown by XPS to disappear after 25 min of broad-band UV–C illumination (λ ≤ 252 nm) at RT, accompanied by a total change of the W charge state from 5+ to 4+. PL investigations have shown that the bleaching of Pr^3+^ emission also occurs under intra 4f-shell excitation due to thermal ionization of the ^1^S_0_ excited state which lies only 7.7 meV below the BPW conduction band edge. Moreover, EPR studies have shown that electrons ionized from Pr^3+^ are also captured by deep interstitial oxygen traps, thus proving that the charge-transfer phenomenon proceeds via the conduction band.

As Raman and XRD spectra show, BPW is stable up to at least 870 K in an inert atmosphere but oxidizes when heated in air above 550 K, decomposing to BaWO_4_ and PrO_2–*x*_. This was confirmed by DSC and TGA measurements. Additionally, considering the presence of interstitial oxygen which can also contribute to gradual Pr and W-oxidation (while exposed to potent ionizing radiation), it makes the material hardly suitable for most applications without a protective coating.

## Supporting Information Available

X-ray diffraction, XAS reference data, PL, calculated electronic structures, DSC and TGA results, etc. (PDF)

## Abbreviations

UV: ultraviolet
DPs: double perovskites
TM: transition metals
RE: rare-earth (ions or atoms)
BCW: Ba_2_CeWO_6_ double perovskite
BPW: Ba_2_PrWO_6_ double perovskite
XRD: X-ray diffraction
XPS: X-ray photoelectron spectroscopy
XAS: X-ray absorption spectroscopy
SG: space group
FTIR: Fourier transform infrared (spectroscopy)
EPR: electron paramagnetic resonance
SEM: scanning electron microscopy
fwhm: full width at half-maximum
CCD: charge-coupled device
NA: numerical aperture
RT: room temperature
PL: photoluminescence
PLE: photoluminescence excitation
NIR: near-infrared
LT: low temperature
HP: high pressure
DAC: diamond anvil cell
ICSD: Inorganic Crystal Structure Database
CCDC: Cambridge Crystallographic Data Centre
JCPDS: Joint Committee on Powder Diffraction Standards
BE: binding energy
O_i_: interstitial oxygen
NUV: near-ultraviolet
HT: high temperature
DSC: differential scanning calorimetry
TGA: thermogravimetric analysis
