# Supplementary material for: Synthesis and Properties of the Ba2PrWO6 Double Perovskite
Source: Inorg Chem. 2024 May 20;63(22):10194–206. doi: 10.1021/acs.inorgchem.4c00567 (PMC11151198; doi:10.1021/acs.inorgchem.4c00567)
Supplement: Supplementary file 1 — ic4c00567_si_001.pdf [file ic4c00567_si_001.pdf]

Supporting material

Synthesis and properties of the Ba_2PrWO_6 double perovskite

*Damian Wlodarczyk[1], Mikolaj Amilusik[2], Katarzyna M. Kosyl[1], Maciej Chrunik[3], Krystyna Lawniczak-Jablonska[1], Hanka Przybylinska[1], Paulina Kosmela[4], Michal Strankowski[4], Lev-Ivan Bulyk[1], Volodymyr Tsiumra[1], Rajibul Islam[1,5], Carmine Autieri[1,6], Fei Xue[5], Marcin Zajac[7], Anastasiia Lysak[1], Roman Minikayev[1], Michal Bockowski[2], Andrzej Suchocki[1]

[1]Institute of Physics, Polish Academy of Sciences, Aleja Lotników 32/46, PL-02668, Warsaw, Poland

[2]Institute of High Pressure, Polish Academy of Sciences, Sokołowska 29/37, PL-01142, Warsaw, Poland

[3]Military University of Technology, Gen. Sylwestra Kaliskiego 2, PL-00908, Warsaw, Poland

[4]Gdansk University of Technology, G. Narutowicza 11/12, PL-80233, Gdansk, Poland

[5]Department of Physics, University of Alabama at Birmingham, 2nd Ave. 1720, AL-35294, South Birmingham, USA

[6]Consiglio Nazionale delle Ricerche CNR-SPIN, UOS Salerno, C. S. V. Ferreri 12, IT-84084 Fisciano (Salerno), Italy

[7]Solaris Synchrotron NSRC, Jagiellonian University, Czerwone Maki 98, PL-30392, Cracow, Poland

Corresponding author email: wlodar@ifpan.edu.pl

Keywords: perovskites; synthesis; crystallographic structure; X-ray spectroscopy, Raman spectroscopy

TABLE OF CONTENT:

3. RESULTS & DISCUSSION S3

3.1 Macro- and SEM microphotographs S3

Figure S1 – photograph of BPW pellet superheated in air S3

3.2 Powder X-ray diffraction S4

Figure S2 – Rietveld refined fit to experimental diffractogram assuming the *R*-3 space group for Ba_2PrWO_6 and the crystal structure S4

Table S1 – refinement parameters for the *R*-3 space group S5

Table S2 – atomic coordinates and occupancy factors S5

3.3 Room temperature XPS data S5

Figure S3 – XAS spectra of Pr in BPW, Pr_6O_{11} and Pr_2O_3 S5

3.4 Charge transfer processes S6

Figure S4 – full range low temperature PL spectrum S6

Figure S5 – calculated electronic band structure S7

3.5 Raman & FTIR Spectroscopies S9

Table S3 – group theory calculations for BPW S9

Figure S6 – PrO_{2-x} impurities on Raman spectra S10

3.6 Material stability at high temperatures S10

Figure S7 – DSC, TGA & Heat capacity data of BPW in air and N_2 S11

4. CONCLUSIONS S13

Figure S8 – theoretical phase transition schemes S13

Table S4 – product placement of the investigated compound among DPs S14

Table S5 – summary of all standardized CIF data S15

Addendum: the section numbers here correspond to those in the main text, where the supporting figures and tables were mentioned.

3. RESULTS AND DISCUSSION

3.1 Macro- and SEM microphotographs.

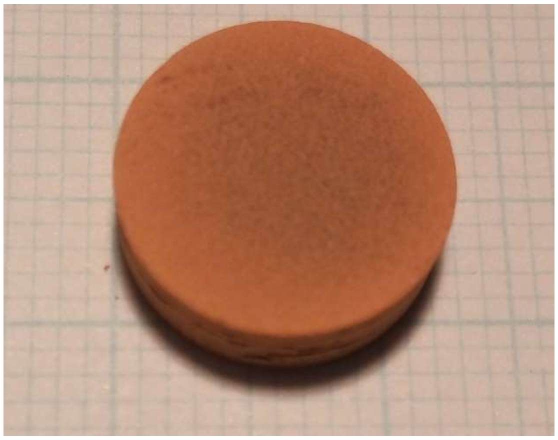

Figure S1. Photograph of BPW heated in air up to 600°C. BPW decomposes mainly into PrO_{2-x}, $BaWO_4$, and $BaPr_2WO_7$.

3.2 Powder X-ray diffraction.

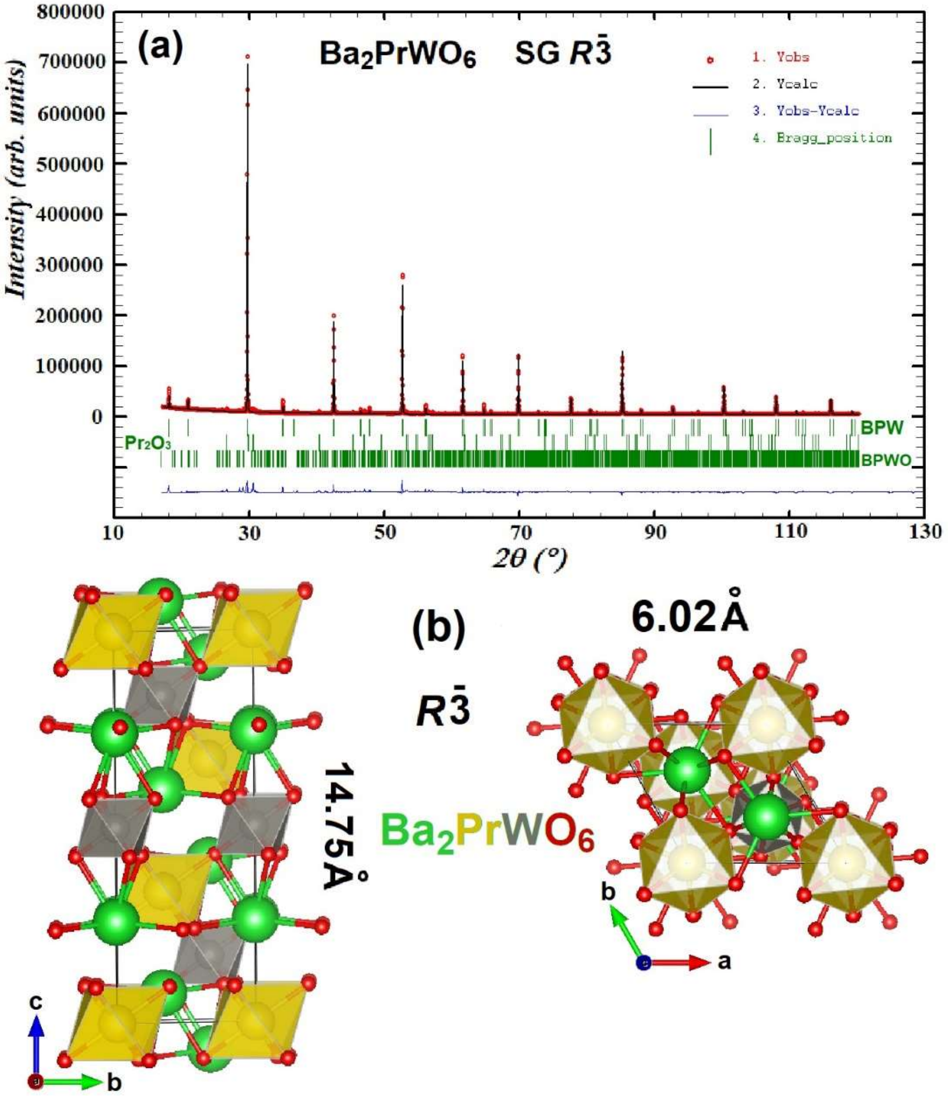

Figure S2. (a) Experimental XRD pattern of the synthesis product (red dots). The black line is the Rietveld refined fit assuming 95.62% of the Ba_2PrWO_6 *R*-3 phase, 3.98% of $BaPr_2WO_7$ (BPWO), and 0.4% of Pr_2O_3. The blue line is the difference between experimental and calculated data. Green bars denote Bragg positions of the three phases. (b) Projection of the unit cell on the *bc* plane (left) and the *ab* plane (right). Green, yellow, grey, and red spheres denote barium, praseodymium, tungsten, and oxygen ions, respectively.

Table S1. Rietveld reliability factors for the alternative *R*-3 SG.

Formula	SG	Z	V [Å^3]	d_{cal} [g/cm^3]	R_B	R_P	R_{WP}	R_{EXP}	N_σ GoF	χ2	Fract [%]
Ba_2PrWO_6	*R*-3	3	463.261	7.478	6.02						95.62
$BaPr_2WO_7$	*P*2$_1$/*b*	4	623.894	7.612	28.7	18.5	17.5	2.66	2736.675	43.1	3.98
Pr_2O_3	*P*-3*m*	1	77.682	7.050	57.9						0.40

Table S2. Atomic coordinates and occupancy factors for the *R*-3 space group.

Site label	x/*a*	y/*b*	z/*c*	occupancy
Ba1	0.514(2)	0.00000	0.25968(6)	1.00000
Pr1	0.00000	0.00000	0.00000	1.00000
W1	0.00000	0.00000	0.50000	1.00000
O1	0.5170(19)	-0.0463(15)	0.2400(9)	1.00000

3.3 Room temperature XPS data.

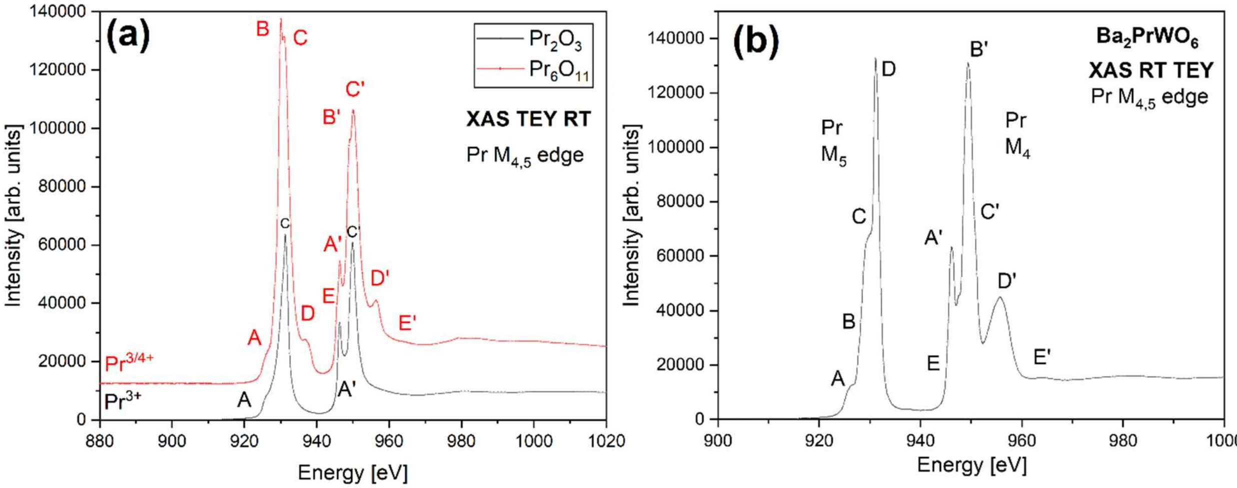

Figure S3. X-ray absorption spectra of praseodymium oxide reference materials (a) and the synthesis product BPW (b).

3.4. Charge transfer processes.

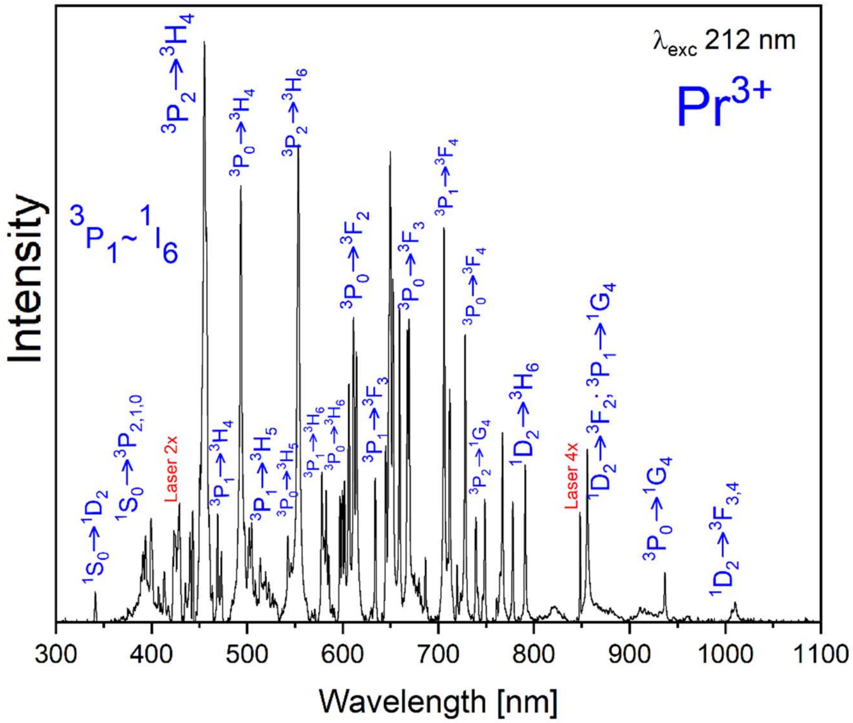

Figure S4. LT PL spectrum recorded without a 435 nm cut-off filter. The second and fourth harmonics of the laser line are marked in red.

Electronic structure calculations were performed with VASP software by using density functional theory projected into the framework of augmented wave method & and Wannier equations [G. Kresse and J. Furthmuller, *Efficient iterative schemes for ab initio total-energy calculations using a plane-wave basis set*, Phys. Rev. 1996, **B54**, 11169]. The relativistic effects were considered consistently and a plane-wave energy cut-off around 650 eV was implemented. As an exchange-correlation functional, we use the generalized gradient approximation (GGA) method [J. P. Perdew, K. Burke and M. Ernzerhof, Generalized gradient approximation made simple, Phys. Rev. Lett. 1996, **77**, 3865]. We have also used the experimentally constructed CIF structures (from refined XRD files) for our calculations and the total energy was converged to 10^{-8} eV with the Gaussian-smearing method. We performed the calculations using 12×12×12 Γ−centered k-mesh with 476 k-points in the Brillouin zone.

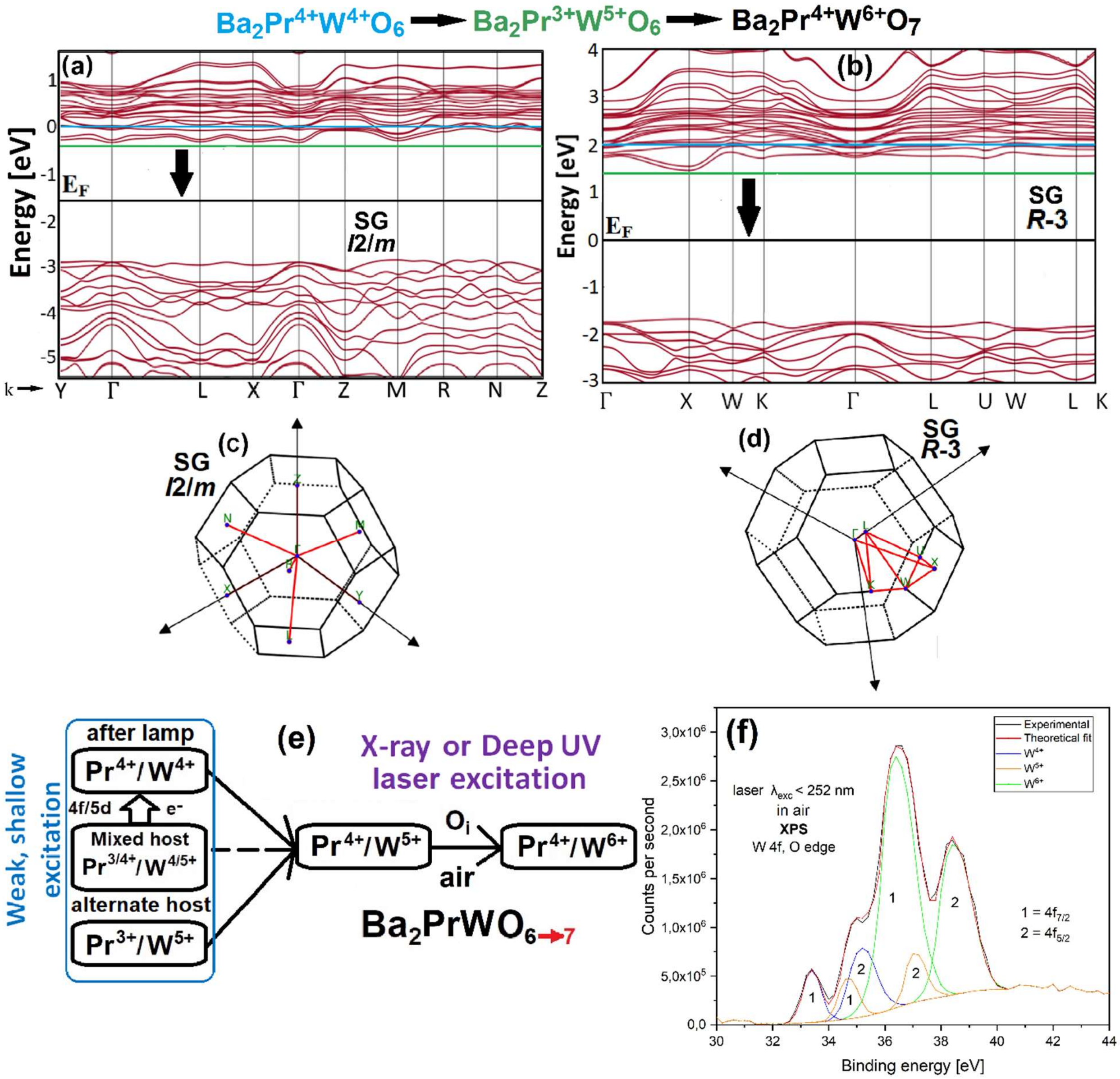

Figure S5. Calculated electronic band structures of $Ba_2Pr^{4+}W^{4+}O_6$ (in red) for *I*2/*m* (a) and *R*-3 (b) space groups. High symmetry points in respective Brillouin zones are shown in (c) & (d). The blue lines (a,b) denote the calculated Fermi level (E_F) positions for $Ba_2Pr^{4+}W^{4+}O_6$. Green and black lines indicate the estimated E_F positions in $Ba_2Pr^{3+}W^{5+}O_6$ and $Ba_2Pr^{4+}W^{6+}O_7$, respectively. The chain of ionic changes between each aforementioned compound under different illumination conditions is depicted in scheme (e). W 4f core state XPS spectra (f) of the sample showcased in Figure 7(b) measured later, under laser UV illumination in the air ($\lambda \leq 252$ nm). Power of the laser was set to 1 mJ.

It can be seen in Figure S5 that the Fermi level position depends strongly on the oxidation state of tungsten, indicating metallic character of the material for W^{4+}, through semiconducting for W^{5+}, to insulating for $Ba_2Pr^{4+}W^{6+}O_7$. When exposed to a more potent energy source (like a UV-C laser light instead of the Xe lamp) it could gradually oxidize the sample from initially obtained W^{4+} or $W^{4+/5+}$ state, towards W^{6+}. The reason behind this mechanism might be an effect of two competing phenomena - thermochemical oxidation of the lattice versus classical charge transfer process. The former phenomenon (simulating O-O bond breakage in the ozone layer) can overwhelm the latter in the air, however local reintegration w O_i from the lattice is also possible although insignificant. Details about such reactions can be examined thoroughly by visiting the Material Stability section 3.6 below. Therefore, extra caution should be taken regarding potential overheating and eventual air leakages during measurements or exploitation – possibly with the application of efficient cooling systems and inert or vacuum conditions, especially considering the elevated absorption rates stemming from the dark coloring of the material. However, we note that the theoretically predicted metallic and semiconducting character of BPW does not fully agree with our experimental findings – they both seem to be greatly underestimated.

3.5. Raman & FTIR Spectroscopies.

Table S3. Group theory analysis for possible space groups assigned to BPW.

Material (SG)	Ion	Wyckoff Positions	Point Symmetry	Representation
**BPW** ***I*2/*m*** ***(C_{2h})***	A^{2+} (Ba)	4i	C_s	$2A_{1g}+A_u+B_g+2B_u$
	$B^{3+/4+}$ (Pr)	2a	C_{2h}	A_u+2B_u
	$B'^{4+/5+}$ (W)	2d	C_{2h}	A_u+2B_u
	O_1^{2-}	4i	C_s	$2A_{1g}+A_u+B_g+2B_u$
	O_2^{2-}	8j	C_1	$3A_{1g}+3A_u+3B_g+3B_u$
$\Gamma_{TOTAL} = 7A_{1g}+7A_u+5B_g+11B_u$; $\Gamma_{ACOUSTIC} = A_u+2B_u$; $\Gamma_{IR} = 6A_u+9B_u$; $\Gamma_{Raman} = 7A_{1g}+5B_g$				
**BPW** ***R*-3** ***(C_{3i})***	A^{2+} (Ba)	2c	C_3	$A_g+A_u+{}^1E_u+{}^2E_u+{}^1E_g+{}^2E_g$
	$B^{3+/4+}$ (Pr)	1a	C_{3i}	$A_u+{}^1E_u+{}^2E_u$
	$B'^{5+/4+}$ (W)	1b	C_{3i}	$A_u+{}^1E_u+{}^2E_u$
	O^{2-}	6f	C_1	$3A_g+3A_u+3{}^1E_u+3{}^2E_u+3{}^1E_g+3{}^2E_g$
$\Gamma_{TOTAL} = 4A_g+6A_u+6{}^1E_u+6{}^2E_u+4{}^1E_g+4{}^2E_g$; $\Gamma_{ACOUSTIC} = A_u+{}^1E_u+{}^2E_u$; $\Gamma_{IR} = 5A_u+5{}^1E_u+5{}^2E_u$; $\Gamma_{Raman} = 4A_g+4{}^1E_g+4{}^2E_g$				

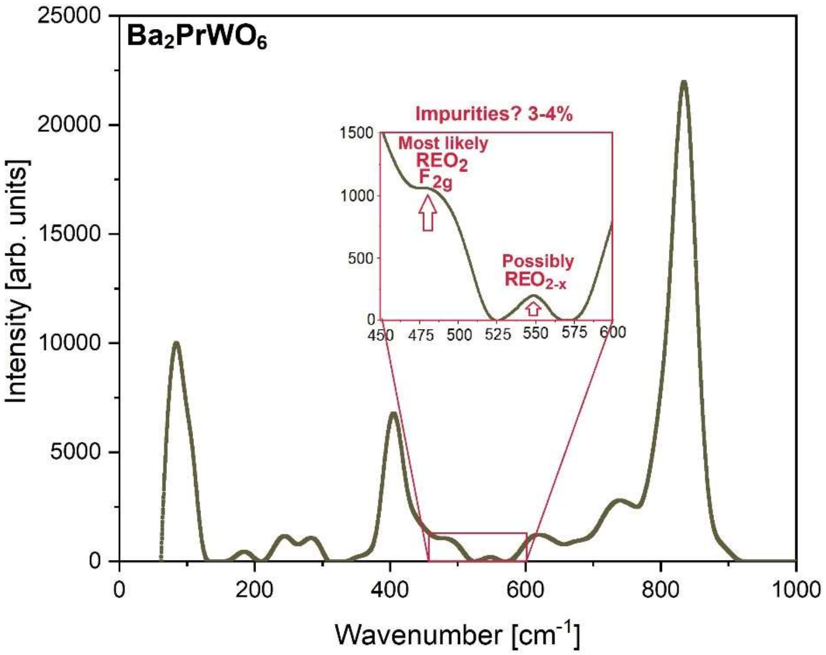

Figure S6. Raman spectra of most common impurity signals from rare-earth tungstate double perovskites also previously found in BCW. [*Synthesis Attempt and Structural Studies of Novel A_2CeWO_6 Double Perovskites (A^{2+} = Ba, Ca) in and Outside of Ambient Conditions'*. *ACS Omega* **2022**, *7* (22), 18382–18408]. In BPW they are PrO_{2-x} analogs of CeO_{2-x} ($0<x<0.5$) species.

3.6. Material stability at high temperatures.

Differential Scanning Calorimetry (DSC) & Heat Capacity (Cp) were taken on Netzsch Phoenix DSC apparatus, model DSC 204 F1. Temperature scans ranged from room temperature to 873K at a heating rate of 10 K per min. A three-stage, heating–cooling–heating system was set in inert-gas/air conditions to assess the behavior the samples in both environments. The heat capacity (C_p) reference was sapphire. Crucibles hosting investigated powders were made out of concave Al pans.

Thermogravimetry (TG) was subsequently carried out in nitrogen and air conditions - 20 ml per min. of nitrogen flow – using Netzsch Tarsus, model TG 209 F3 at temperatures ranging from 293 to 1273 K. The heating/cooling rate was roughly 10 K per min. The mass of each sample placed in a small corundum crucible was approximately 10 mg.

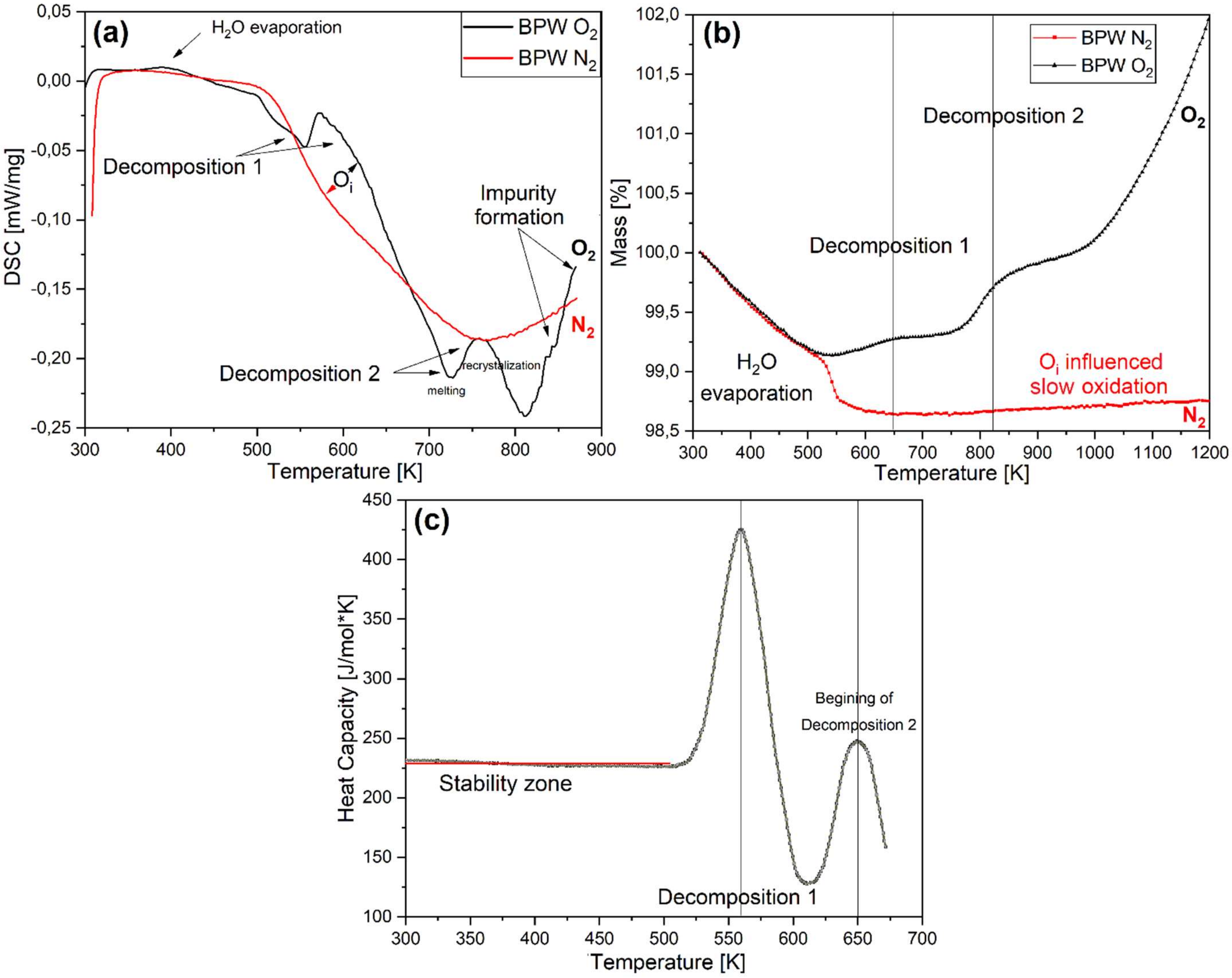

Figure S7. Differential scanning calorimetry (a) and thermogravimetry (b) measurements of Ba_2PrWO_6 (BPW) in the air and in inert N_2 gas at high-temperatures showing decomposition of the material in the former medium. (c) Heat capacity measured against a sapphire reference. The average Cp was calculated in the air-stable zone marked with a red, horizontal line.

There are complementary changes occurring in the temperature ranges denoted as “Decomposition” 1 & 2 in all data presented in Figure S7. Together with the data about minor phases (Pr_2O_3 + PrO_{2-x}, $BaPr_2WO_7$, $BaWO_4$) obtained from HT XRD patterns one can explain the reactions taking place during heating.

The first reaction occurring at temperatures up to about 400 K is dehydration:

$Ba_2PrWO_6 \times n\ H_2O \rightarrow n\ H_2O\ (g) + Ba_2PrWO_6$ accompanied by a loss of mass. The situation drastically changes above ~550 K. In the air, the calorimetry curve (a) has a huge exothermic dip indicating oxidation and in (b) a mass gain is visible. The heat capacity shows a peak.

Decomposition 1 stage occurs at ~ 550 K and is related to Pr^{3+} oxidation:

$$Pr^{3+} \rightarrow Pr^{4+}\ ;\ 2Pr_2O_3 + O_2 \rightarrow 4\ PrO_2\ ;\ 3\ Pr_2O_3 + O_2 \rightarrow Pr_6O_{11}\ (6\ PrO_{2-x});$$

$$2\ Pr_6O_{11} + O_2\ (\text{if } O_i + O_{2-x}) \rightarrow 12\ PrO_{2(-x)}\ ;\ 2\ Ba_2Pr^{3+}W^{5+}O_6 \rightarrow 2\ PrO_2 + 2\ Ba_2WO_4$$

Decomposition 2 occurs at much higher temperatures (~ 750 K) and is related mostly to tungsten oxidation:

$$W^{4/5+} \rightarrow W^{6+}\ ;\ 2\ WO_2 + O_2 \rightarrow 2\ WO_3\ ;\ 2\ W_{18}O_{49}\ (W^{5/6+}O_{3-x}) + 5\ O_2 \rightarrow 36\ W^{6+}O_3$$

$$2\ Ba_2Pr^{4+}W^{4+}O_6 + O_2 \rightarrow 2\ PrO_2 + 2\ Ba_2WO_5\ ;\ 2\ Ba_2WO_4 + O_2 \rightarrow 2\ Ba_2WO_5$$

After the second decomposition stage the amount of some products increases due to overcoming of activation energy barriers:

$$Ba_2WO_5 \rightarrow BaWO_4 + BaO^*\ ;\ BaO^* + Pr_2O_3 + WO_3 \rightarrow BaPr_2WO_7$$

It is worth noting that in inert N_2 gas, there are still some changes occurring within BPW. Mainly, there is still a dip in the red curve in Figure S7(a) although without any pronounced features. In TG (b), the mass also slightly increases. This can be related to gradual oxidation of Pr and W ions due to interstitial oxygen. The average molar heat capacity (Cp) of BPW in the stable region in the air (below 500 K) is 228.2 ± 1.5 J/(mol×K). Median: ~ 227.6 J/(mol×K). In this range Cp (Figure S7(c)) remains stable since the matrix does not change chemically.

4. CONCLUSIONS

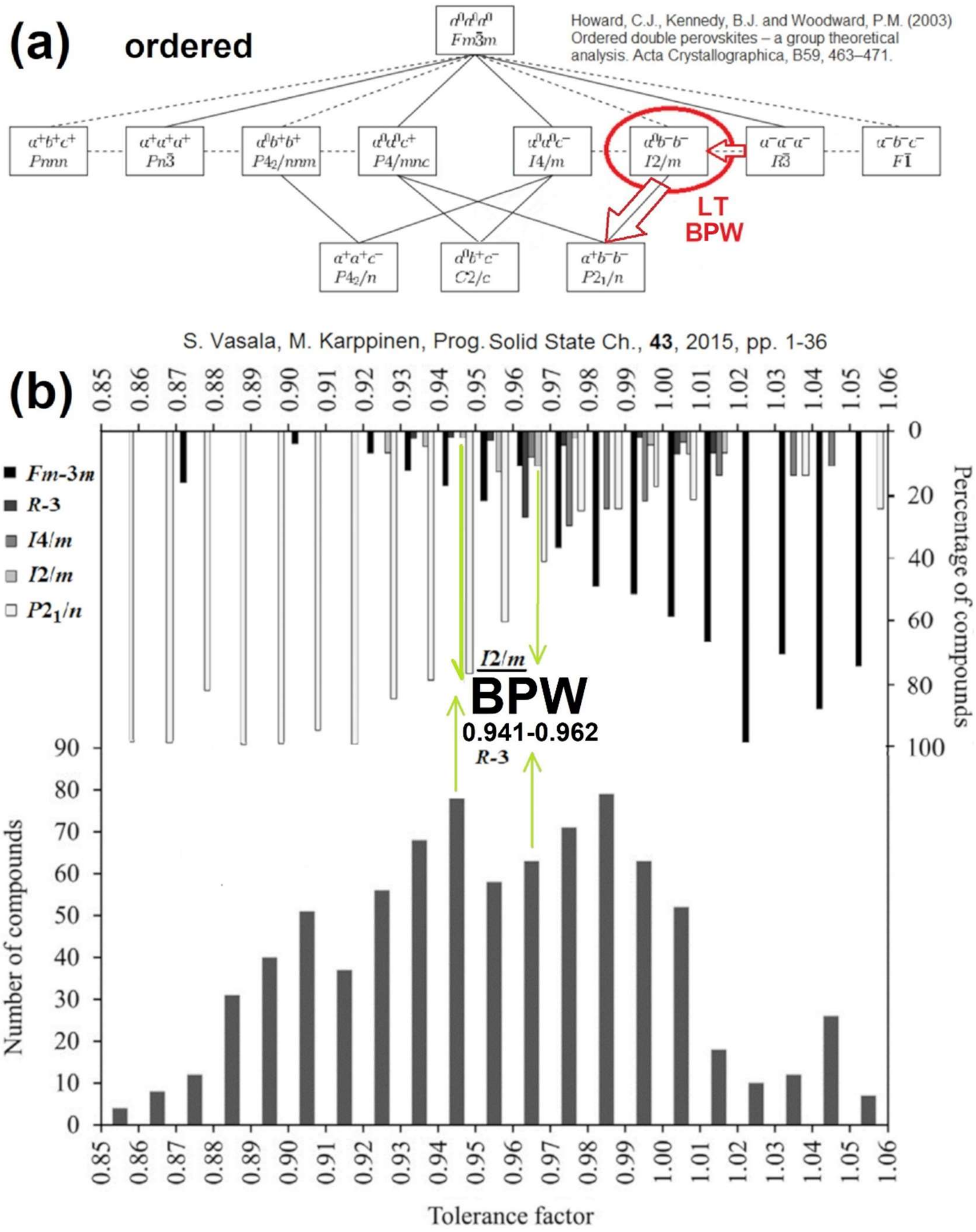

Figure S8. (a) Group–subgroup relations associated with particular tilt systems in ordered double perovskites $A_2BB'X_6$ evaluated by Howard et. al. using group theory. Continuous black lines are first-, and dashed second-order transformations. (b) Possible sample placement.

The first graph was adapted and reprinted with permissions from Howard C. J.; Kennedy B. J.; Woodward P. M. Ordered double perovskites – a group-theoretical analysis, *Acta Crystallogr.* **2003**, *B59*, 463-471. Copyrighted and licensed by *IUCr Journals* Publisher via *Acta Crystallographica B* journal after 2003. Figure S8(b) was licensed from Vasala S.; Karppinen M. $A_2B'B''O_6$ perovskites: A review, *Prog. Solid State Chem.* **2015**, *43* (1-2), 1-36 copyrighted by *Elsevier* Publisher via *Progress in Solid State Chemistry*.

Table S4. Most common space groups and tilt systems, for ordered double-perovskites reported till 2015 by Vasala and Karppinen with addition of our newly synthesized material. The red color depicts our final assignment, green (with asterisks) is a possible option. This table was adapted and reprinted in part with permission from Vasala S.; Karppinen M. $A_2B'B''O_6$ perovskites: A review, *Prog. Solid State Chem.* **2015**, *43* (1-2), 1-36. Copyrighted and licensed by *Elsevier Publisher* via *Progress in Solid State Chemistry* journal since the year 2015.

Glazer tilt	Space Group		Number of compounds	
$a^0a^0a^0$	*Fm*-3*m*		146	
$a^-a^-a^-$	*R*-3*	+1	19*	
$a^0a^0c^-$	*I*4/*m*		44	
$a^0a^0c^+$	*P*4/*mnc*		0	Ordered Perovskites **(BPW)**
$a^0b^-b^-$	*I*2/*m*		19+1	
$a^+b^-b^-$	*P*2$_1$/*n*		310	
$a^-b^-c^-$	*I*-1		2	

Table S5. Standardized CIF data determined from Rietveld refined Ba_2PrWO_6 powder XRD diffractograms for both applicable SGs (*I*2/*m* & *R*-3).

**Chemical formula**	Ba_2PrWO_6									
**Analytical formula**	$Ba_{1.96}Pr_{1.05}WO_{6.05}$									
**Formula weight [g/mol]**	695.404									
**Melting Point [K]**	1623									
**Source**	X-ray laboratory									
**Temperature [K]**	298									
**Pressure [atm]**	1									
**Wavelength [A]**	1.5406 (Cu Kα)									
**Crystal System**	Monoclinic					Rhombohedral				
**SG No.**	12					148				
**Dimensions**	a	b	c	α=γ	β	a	b	c	α=β	γ
**Size [A]**	6.0219(3)	6.0218(3)	8.5167(3)	90	90.001(3)	6.0219(4)	6.0219(4)	14.7509(8)	90	120
**V [A^3]**	308.843					463.261				
**Z**	2					3				
**d [g/cm^3]**	7.479					7.478				
**χ2**	42.2					43.1				
**R_p**	18.3					18.5				
**R_{wp}**	17.3					17.5				
**R_B**	28.5					28.7				
**R_{EXP}**	2.66					2.66				
**GoF**	2674.3					2736.7				
